# RNA Methylome Reveals the m^6^A-mediated Regulation of Flavor Metabolites in Tea Leaves under Solar-withering

**DOI:** 10.1016/j.gpb.2023.02.003

**Published:** 2023-02-14

**Authors:** Chen Zhu, Shuting Zhang, Chengzhe Zhou, Caiyun Tian, Biying Shi, Kai Xu, Linjie Huang, Yun Sun, Yuling Lin, Zhongxiong Lai, Yuqiong Guo

**Affiliations:** 1 College of Horticulture, Fujian Agriculture and Forestry University, Fuzhou 350002, China; 2 Institute of Horticultural Biotechnology, Fujian Agriculture and Forestry University, Fuzhou 350002, China; 3 Tea Industry Research Institute, Fujian Agriculture and Forestry University, Fuzhou 350002, China

**Keywords:** *Camellia sinensis*, RNA methylation, Epitranscriptome, Secondary metabolite, Withering

## Abstract

The epitranscriptomic mark *N*^6^-methyladenosine (m^6^A), which is the predominant internal modification in RNA, is important for plant responses to diverse stresses. Multiple environmental stresses caused by the tea-**withering** process can greatly influence the accumulation of specialized metabolites and the formation of tea flavor. However, the effects of the m^6^A-mediated regulatory mechanism on flavor-related metabolic pathways in tea leaves remain relatively uncharacterized. We performed an integrated RNA methylome and transcriptome analysis to explore the m^6^A-mediated regulatory mechanism and its effects on flavonoid and terpenoid metabolism in tea (*Camellia sinensis*) leaves under solar-withering conditions. Dynamic changes in global m^6^A level in tea leaves were mainly controlled by two m^6^A erasers (CsALKBH4A and CsALKBH4B) during solar-withering treatments. Differentially methylated peak-associated genes following solar-withering treatments with different shading rates were assigned to terpenoid biosynthesis and spliceosome pathways. Further analyses indicated that CsALKBH4-driven RNA demethylation can directly affect the accumulation of volatile terpenoids by mediating the stability and abundance of terpenoid biosynthesis-related transcripts and also indirectly influence the flavonoid, catechin, and theaflavin contents by triggering alternative splicing-mediated regulation. Our findings revealed a novel layer of epitranscriptomic gene regulation in tea flavor-related metabolic pathways and established a link between the m^6^A-mediated regulatory mechanism and the formation of tea flavor under solar-withering conditions.

## Introduction

As the predominant internal modification in eukaryotic mRNAs, *N*^6^-methyladenosine (m^6^A) is a critical transcriptional regulator in a novel molecular mechanism that profoundly affects various biological processes by modulating multiple aspects of mRNA processing and metabolism [1], including mRNA abundance [2], [3], stabilization [4], and splicing [5]. Along with the well-studied epigenetic regulation via modifications to DNA and histones, the dynamic and reversible m^6^A modification is mediated by m^6^A methyltransferases (writers) and demethylases (erasers). In addition to these two crucial components, m^6^A readers are responsible for the localization and recognition of m^6^A-containing RNA sequences, which are necessary for the implementation of the biological effects of m^6^A modifications. Consequently, m^6^A writers, erasers, and readers collaboratively orchestrate a complex regulatory network that governs m^6^A modifications. Although there has been considerable progress in the research conducted to clarify the potential functions of m^6^A modifications in animals, the effects of the m^6^A regulatory mechanism in the plant kingdom are just beginning to be determined. The m^6^A-mediated regulatory mechanism reportedly influences the normal growth of *Arabidopsis thaliana* roots and shoots [6], [7]. Furthermore, there is growing evidence that m^6^A modifications are also involved in regulating responses to diverse environmental stresses, including drought [8], cold [9], and ultraviolet (UV) radiation [10]. More recently, a few studies have started to precisely functionally characterize the m^6^A regulatory mechanism or m^6^A regulatory genes in the plant kingdom [11], [12], [13], [14]. Compared with model plants, there has been relatively little research on the regulatory mechanisms as well as the functions of m^6^A modifications in horticultural crops.

Tea (*Camellia sinensis*) is an important and traditional economically valuable crop cultivated on a large scale in many developing and developed countries. Its tender buds and leaves are mostly used to produce highly consumed and popular beverages. Oolong tea, which is one of the six tea types in China, is famous for its elegant floral and fruity aroma, as well as its unique brisk-smooth and mellow taste. The development of the flavor and sensory characteristics of oolong tea largely depends on the postharvest manufacturing process [15]. During the manufacturing of oolong tea, withering is the first essential stage affecting the palatability and commercial value of the final product. The harvested leaves are still in a live state and continue to be exposed to various environmental stresses during the withering stage, in which there are obvious changes to multiple tastes and aroma compounds, as well as endogenous phytohormones, which directly or indirectly endow oolong tea with its characteristic flavor and health benefits [16], [17], [18]. Among these taste compounds in tea plants, flavonoids are a large group of secondary metabolites that are closely associated with tea palatability. More specifically, catechins are the dominant flavonoid components and comprise 12%–24% of the dry weight in tea leaves [19]. Flavonoids and catechins are the major contributors to the astringency and bitterness of tea [20]. Moreover, catechins can be further oxidized to high-molecular-weight polymeric compounds (*i.e.*, theaflavins) that provide tea with beneficial health-promoting properties and a characteristic mellow taste [21]. As the most representative aroma compounds in oolong tea, volatile terpenoids are critical components of high-quality oolong tea because of their contributions to the pleasant floral and fruity fragrance. Decades of studies have demonstrated that multiple environmental stresses caused by withering treatments induce several multidimensional responses (*e.g.*, at the genetic and metabolic levels) that further modulate the formation of the oolong tea flavor [22], [23]. However, information regarding the upstream mechanism regulating the flavor-related genes and relevant metabolites remains limited.

Previous research has indicated that solar-withering, which is a conventional method for producing tea, may be used to improve the flavor of high-quality oolong tea [24], [25]. Light serves as an energy source and signaling molecule required for the metabolic changes that occur during solar-withering. Typically, light quality and intensity are crucial parameters that determine the effects of solar-withering on tea quality. Earlier studies largely focused on the mechanism underlying the effects of light quality on the tea flavor [26], [27]. Few researchers have investigated the effects of different shading rates on the metabolism of tea flavor compounds and the formation of tea flavors during the solar-withering stage. The establishment of traditional solar-withering methods relies heavily on the subjective experiences of tea producers and random trials, leading to fluctuations in tea quality. To overcome this limitation, characterizing the metabolic pathways that affect flavor formation as well as the regulatory mechanisms under different shading rates during the solar-withering stage is vital for optimizing the shading rate to produce high-quality oolong tea efficiently. There has recently been accumulating evidence that epigenetic changes, including DNA methylation and histone modifications, are crucial for the biosynthesis of secondary metabolites and tea aroma formation [28], [29]. Unfortunately, the effects of RNA methylation, which is another epigenetic modification, and the precise regulatory mechanisms underlying the m^6^A-mediated flavor formation in the tea-withering stage remain largely unknown. The availability of a chromosome-level tea genome has laid a solid foundation for investigations on m^6^A modifications in tea plants [30].

In the present study, an integrated RNA methylome and transcriptome analysis was conducted to elucidate the effects of m^6^A modifications on the formation of flavor-related compounds in tea leaves in response to different shading rates during the solar-withering stage. We systematically identified the differentially methylated peak (DMP)-associated genes and revealed that the changes to m^6^A in many DMP-associated genes are inextricably associated with the solar-withering conditions, especially the shading rate. We further demonstrated that CsALKBH4*-*mediated RNA demethylation alters the extent of the m^6^A modifications within the 3′ untranslated region (3′ UTR) and near the stop codon and regulates the expression levels of m^6^A-modified RNAs, thereby affecting the accumulation of flavor metabolites and tea palatability. The effects of the m^6^A-mediated alternative splicing (AS) regulatory mechanism during the solar-withering stage were also explored. These findings described herein provide important insights into the regulatory effects of m^6^A modifications on tea plants and the contribution of the m^6^A-mediated regulatory mechanism to the development of high-quality oolong tea via a solar-withering method.

## Results

### Significant changes in global abundance and distribution of m^6^A modifications in tea leaves in response to solar-withering treatments

**Figure 1:**
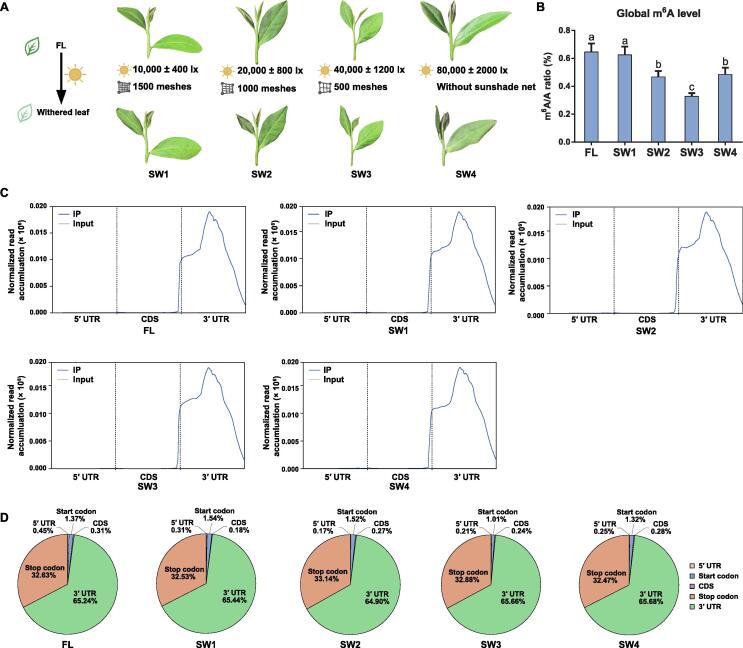
**Global abundance anddistributionof m^6^A modificationsin tea leaves under solar-withering with different shading rates** **A.** The external phenotypes of withered leaves before and after solar-withering with different shading rates. **B.** Dynamics of global m^6^A level in tea leaves under solar-withering with different shading rates. Data are presented as mean ± SD. Different lowercase letters over the error bars indicate significantly different groups via one-way ANOVA and Tukey’s *post hoc* test (*P* < 0.05). **C.** Metagenomic profiles of m^6^A distribution along transcripts. **D.** Pie charts showing the fractions of m^6^A peaks falling into five transcript segments (5′ UTR, start codon, CDS, stop codon, and 3′ UTR). m^6^A, *N*^6^-methyladenosine; IP, immunoprecipitation; FL, fresh leaf; SW1, solar-withering with a high shading rate; SW2, solar-withering with a middle shading rate; SW3, solar-withering with a low shading rate; SW4, solar-withering with natural sunlight; 5′ UTR, 5′ untranslated region; CDS, coding sequence; 3′ UTR, 3′ untranslated region; SD, standard deviation; ANOVA, analysis of variance.

**Supplementary Figure S1:**
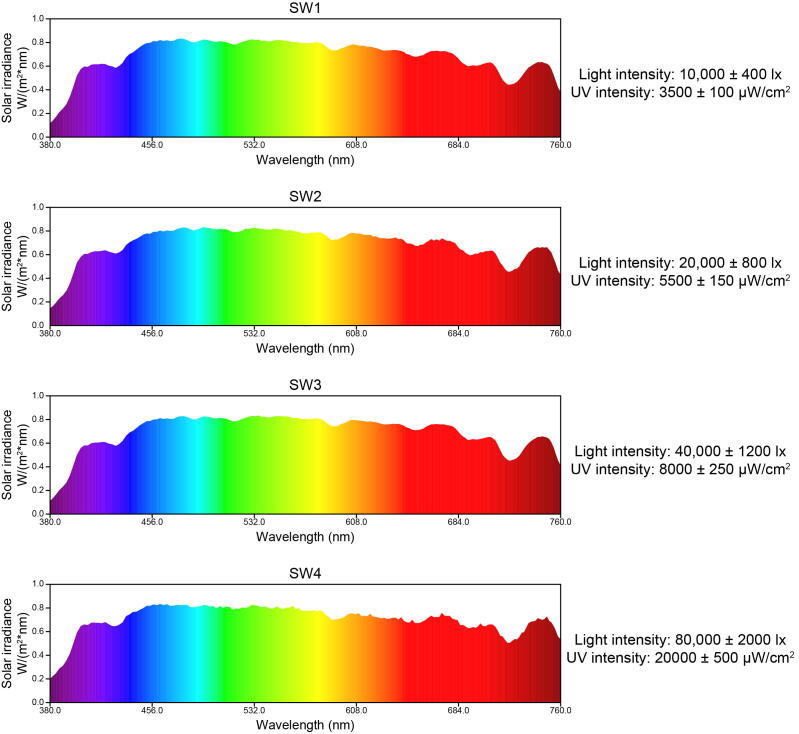
**The light intensity, spectrum, and UV intensity of solar-withering with different shading rates** SW1, solar-withering with a high shading rate; SW2, solar-withering with a middle shading rate; SW3, solar-withering with a low shading rate; SW4, solar-withering with natural sunlight; UV, ultraviolet.

The light intensity, spectrum, and UV intensity of solar-withering treatments with different shading rates were monitored (Figure S1). The withering treatments were performed as follows: solar-withering with a high shading rate (SW1), solar-withering with a moderate shading rate (SW2), solar-withering with a low shading rate (SW3), and solar-withering with natural sunlight (SW4). Notably, the light intensity and UV intensity under the sunshade net decreased as the shading rate increased. However, the use of the sunshade net had little effect on the spectral composition, light quality, and wavelength. There were considerable differences in the UV intensity between the solar-withering with and without the shading net. The shading treatment substantially decreased the UV intensity. From SW1 to SW3, the different shading rates had relatively little effect on the UV intensity. The external characteristics of fresh leaves (FLs) and the solar-withered leaves produced using different shading rates were recorded (Figure 1A). FLs were straight, glossy, and jade green. Decreases in the shading rate during the solar-withering treatments resulted in increasingly shrunken, deformed, and darker-colored withered leaves. We then calculated the global m^6^A/A ratio of these tea leaves (Figure 1B). The solar-withering treatments with different shading rates decreased the global m^6^A level. The comparison of the various leaves detected a gradually decreasing trend in the overall m^6^A level from FLs to the SW3 leaves, but the m^6^A level rebounded following the SW4 treatment.

### Overview of the m^6^A MeRIP-seq

**Supplementary Figure S2:**
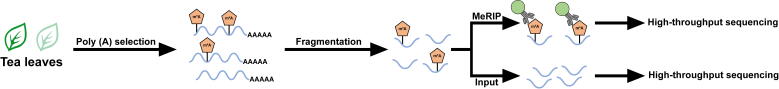
**Brief workflow for MeRIP-seq** The fragmented RNA was divided into two portions, one of which was enriched with an m^6^A-specific antibody in IP buffer. The other RNA without IP treatment was used as the input control. Finally, the two portions of RNA were sequenced using an Illumina NovaSeq 6000 platform. IP, immunoprecipitation; m^6^A, *N*^6^-methyladenosine; MeRIP, m^6^A methylated RNA immunoprecipitation.

**Supplementary Figure S3:**
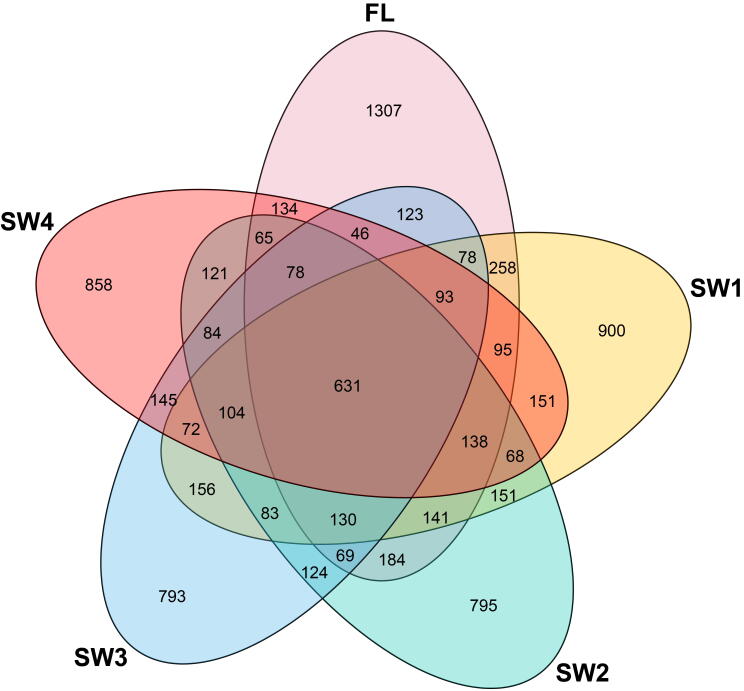
**Venn diagrams showing the overlap of m^6^A peaks identified from fresh leaves and four solar-withered leaves with different shading rates** FL, fresh leaves; SW1, solar-withering with a high shading rate; SW2, solar-withering with a middle shading rate; SW3, solar-withering with a low shading rate; SW4, solar-withering with natural sunlight.

**Supplementary Figure S4:**
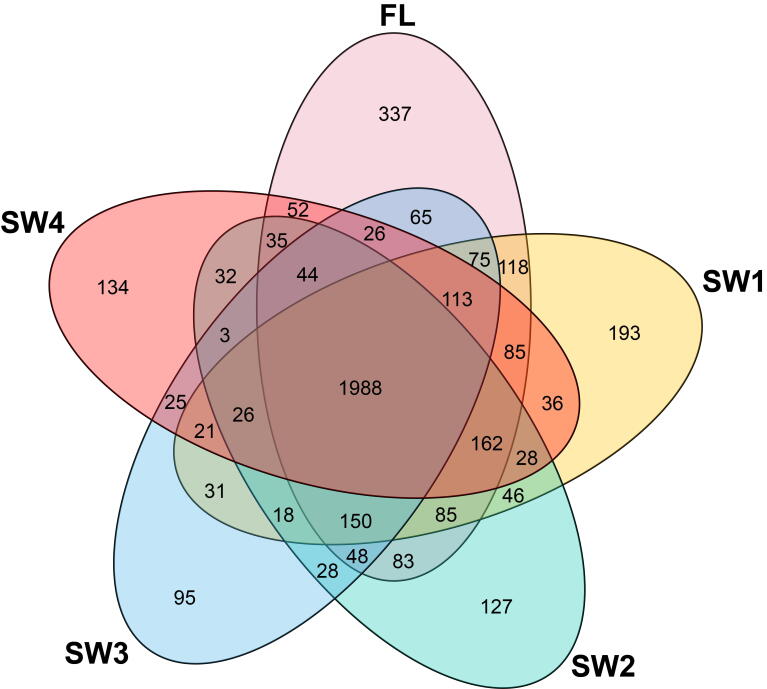
**Venn diagrams showing the overlap of m^6^A-marked genes identified from fresh leaves and four solar-withered leaves with different shading rates** FL, fresh leaves; SW1, solar-withering with a high shading rate; SW2, solar-withering with a middle shading rate; SW3, solar-withering with a low shading rate; SW4, solar-withering with natural sunlight.

**Supplementary Figure S5:**
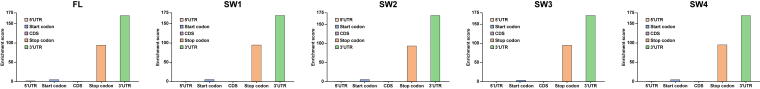
**Enrichment score of m^6^A peaks in five transcript segments** 5′ UTR, 5′ untranslated region; CDS, coding sequence; 3′ UTR, 3′ untranslated region; FL, fresh leaves; SW1, solar-withering with a high shading rate; SW2, solar-withering with a middle shading rate; SW3, solar-withering with a low shading rate; SW4, solar-withering with natural sunlight.

To ascertain the effects of m^6^A-mediated regulatory mechanisms under solar-withering conditions, we performed an m^6^A methylated RNA immunoprecipitation sequencing (MeRIP-seq) experiment to analyze the m^6^A modifications at the mRNA level in tea leaves that underwent solar-withering with different shading rates (Figure S2). A total of 34–50 and 63–76 million high-quality clean reads were obtained for the immunoprecipitated (IP) libraries and input libraries, respectively, among which 28–41 and 55–67 million reads were mapped to the tea genome (Table S1). The base quality score of 20 (Q20) and base quality score of 30 (Q30) indices for each library exceeded 93% and 88%, respectively. Only the m^6^A peaks in all biological replicates with high-confidence coefficients were selected for further analyses. A total of 3570, 3249, 2966, 2809, and 2883 high-confidence m^6^A peaks were identified for the FL, SW1, SW2, SW3, and SW4 samples, respectively (Figure S3). Among these m^6^A peaks, 4605 were newly generated after the solar-withering treatment. Additionally, 31,720, 31,958, 32,146, 32,215, and 31,988 transcripts were obtained from the FL, SW1, SW2, SW3, and SW4 samples, respectively. Next, we identified 5277 high-confidence m^6^A peaks in 4289 transcripts. A total of 1988 m^6^A-marked genes were present in all samples (Figure S4). The solar-withering treatment induced the production of 843 new m^6^A-marked genes. The changes in the number of m^6^A-marked genes in response to solar-withering with different shading rates were consistent with the total m^6^A abundance dynamics. These observations suggest that the m^6^A abundance is closely related to these changes in the number of m^6^A-marked genes, which is in accordance with the findings of a previous study on tomato [11]. The number of m^6^A-marked genes gradually decreased from FLs to the SW3 leaves, whereas there were more m^6^A-marked genes in the SW4 leaves than in the SW3 leaves. In addition, most of the m^6^A-marked genes (2229 genes) were common to the FL *vs.* SW2 and FL *vs.* SW4 comparisons, whereas 219 and 216 m^6^A-marked genes were unique to the FL *vs.* SW2 and FL *vs.* SW4 comparisons, respectively. These results imply that the m^6^A-marked genes did not differ significantly between these two comparisons, with most m^6^A-marked genes common to both comparisons. Most of the 4289 m^6^A-marked genes in the five samples (average of 96.90%) contained a single m^6^A peak, with only a few containing more than three m^6^A peaks (Table S2). The distribution of m^6^A peaks in the tea leaves during the solar-withering treatment was then investigated. Major m^6^A modifications within transcripts were primarily near the stop codon and 3′ UTR (Figure 1C). Only 1.46%–2.13% of the m^6^A modifications were located in the coding sequence (CDS) and 5′ UTR or near the start codon (Figure 1D). From FLs to the SW3 leaves, the proportion of m^6^A modifications increased in the 3′ UTR and near the stop codon, but decreased in the 5′ UTR and CDS and near the start codon. Surprisingly, the proportion of m^6^A modifications in the 3′ UTR and around the stop codon was lower in the SW4 leaves than that in the SW3 leaves, whereas the opposite trend was observed for the m^6^A peaks in the 5′ UTR and CDS and near the start codon. Next, the m^6^A peaks were normalized by the enrichment algorithm [31]. The results revealed that m^6^A modifications accumulated preferentially in the 3′ UTR and around the stop codon (Figure S5). Overall, the distribution of m^6^A peaks in tea leaves during the solar-withering treatment changed slightly.

**Supplementary Figure S6:**
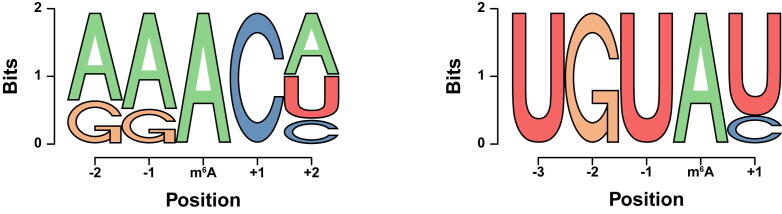
**Top sequence motifs identified within m^6^A peaks in tea leaves under solar-withering with different shading rates** m^6^A, *N*^6^-methyladenosine.

To identify the enriched motifs within the m^6^A peaks in tea plants, all of the m^6^A peaks were scanned using the MEME suite [32] and HOMER tool [33]. As expected, the canonical motif RRACH (R = G/A and H = A/U/C) was enriched in most of the m^6^A peaks (Figure S6). Moreover, another enriched motif, UGUAY (Y = C/U), was similar to the plant-specific motif URUAY, which can be recognized by m^6^A readers [34]. Although the UGUAY motif is relatively common in the m^6^A peaks of tomato [11], it has not been detected in other plant species. Hence, the enriched motifs at m^6^A-modified sites were relatively conserved in two horticultural plant species (*i.e.*, tea and tomato).

### Analysis of DMP-associated genes

To clarify the potential effects of m^6^A modifications during the solar-withering stage, we first searched for DMPs in the m^6^A methylome. The DMPs were identified according to the following criteria: |fold change (FC)| ≥ 2 and *P* < 0.05. A total of 265 DMPs were detected in the FL *vs.* SW1 comparison, of which 203 and 62 were hypermethylated and hypomethylated peaks, respectively (Table S3). The 215 DMPs identified in the FL *vs.* SW4 comparison were similar to the number of DMPs detected in the FL *vs.* SW2 comparison (*i.e.*, 217), but more than the 183 DMPs identified in the FL *vs.* SW3 comparison. These results reflected the apparent changes in the global m^6^A status in the withered leaves following the solar-withering treatments with different shading rates. The number of detected DMPs decreased from the FL *vs.* SW1 comparison to the FL *vs.* SW3 comparison, whereas it increased substantially in the FL *vs.* SW4 comparison. Intriguingly, the change in the number of DMPs under solar-withering conditions was in accordance with the overall change in the total m^6^A level. Hence, the m^6^A modifications in tea leaves were tightly associated with the solar-withering treatment conditions, especially the shading rate.

**Supplementary Figure S7:**
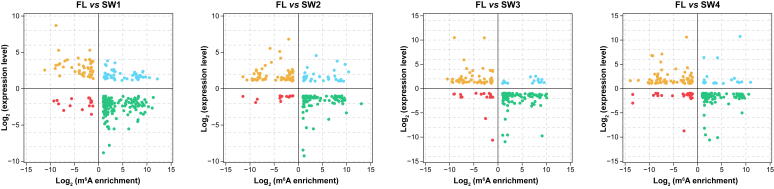
**Correlation between m^6^A modification and expression levels of the DMP-associated genes under solar-withering with different shading rates** DMP-associated genes, differentially methylated peak-associated genes; m^6^A, *N*^6^-methyladenosine; FL, fresh leaves; SW1, solar-withering with a high shading rate; SW2, solar-withering with a middle shading rate; SW3, solar-withering with a low shading rate; SW4, solar-withering with natural sunlight.

To evaluate the overall correlation between m^6^A modifications and gene expression levels in response to solar-withering treatments, 1137 DMP-associated genes with varying expression levels between samples were selected (Figure S7). The results showed that 74.9% of the DMP-associated genes in the FL *vs.* SW1 comparison, 79.6% of the DMP-associated genes in the FL *vs.* SW2 comparison, 83.2% of the DMP-associated genes in the FL *vs.* SW3 comparison, and 79.6% of the DMP-associated genes in the FL *vs.* SW4 comparison with increased or decreased m^6^A levels displayed negatively regulated gene expression. In contrast, only 25.1% of the DMP-associated genes in the FL *vs.* SW1 comparison, 20.4% of the DMP-associated genes in the FL *vs.* SW2 comparison, 16.8% of the DMP-associated genes in the FL *vs.* SW3 comparison, and 20.4% of the DMP-associated genes in the FL *vs.* SW4 comparison with increased or decreased m^6^A levels displayed positively regulated gene expression. Thus, the m^6^A modifications in most DMP-associated genes were negatively correlated with gene expression levels during the solar-withering treatment. Interestingly, combined with the increasing proportion of m^6^A modifications in the 3′ UTR and near the stop codon from FLs to the SW3 leaves, m^6^A modifications within these regions tended to decrease the mRNA abundance. Furthermore, the proportion of m^6^A modifications within the 3′ UTR and around the stop codon was lower in the SW4 leaves than that in the SW3 leaves, which was consistent with the obvious decrease in the proportion of DMP-associated genes with expression levels that were negatively correlated with m^6^A abundance. The m^6^A peaks distributed within the 3′ UTR and around the stop codon were negatively correlated with the mRNA abundance, whereas the m^6^A peaks located in the 5′ UTR and CDS and near the start codon tended to be positively correlated with gene expression. Therefore, the multifaceted effects of m^6^A modifications on mRNA expression may depend on the position of the m^6^A peaks. The specific distribution of m^6^A peaks is closely related to gene expression in tea leaves as well as in several other important crops, including rice [35], strawberry [13], and apple [36].

**Figure 2:**
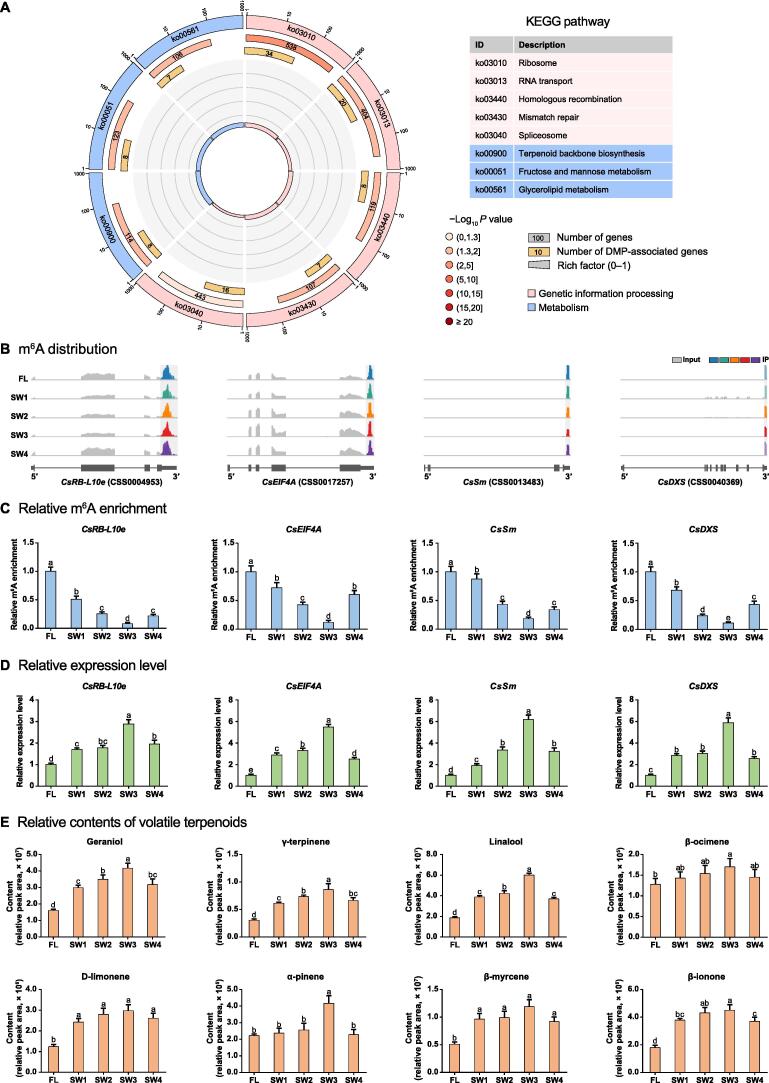
**m^6^A abundances of representative DMP-associated genes werenegatively correlated with their expression levelsas well asthe accumulation of volatile terpenoids under solar-withering with different shading rates** **A.** KEGG enrichment analysis of DMP-associated genes. From the outside to the inside, the first circle indicates enriched KEGG pathways and the number of genes corresponds to the outer circle. The second circle indicates the number of genes in the genome background and the *P* value for the enrichment of the genes in the specified KEGG pathway. The third circle indicates the number of DMP-associated genes. The fourth circle indicates the enrichment factor of each KEGG pathway. **B.** The distribution of m^6^A reads in *CsRB-L10e*, *CsEIF4A*, *CsSm*, and *CsDXS*. Exons and introns in the gene structures are represented by thick boxes and lines, respectively. **C.** Relative m^6^A enrichment of *CsRB-L10e*, *CsEIF4A*, *CsSm*, and *CsDXS* under solar-withering with different shading rates determined by m^6^A-IP-qPCR. **D.** Relative expression levels of *CsRB-L10e*, *CsEIF4A*, *CsSm*, and *CsDXS* under solar-withering with different shading rates determined by qRT-PCR. **E.** Relative contents of volatile terpenoids under solar-withering with different shading rates determined by GC–MS. Data are presented as mean ± SD. Different lowercase letters over the error bars indicate significantly different groups via one-way ANOVA and Tukey’s *post hoc* test (*P* < 0.05). DMP, differentially methylated peak; KEGG, Kyoto Encyclopedia of Genes and Genomes; m^6^A-IP-qPCR, m^6^A-immunoprecipitation-quantitative polymerase chain reaction; qRT-PCR, quantitative real-time polymerase chain reaction; GC–MS, gas chromatography–mass spectrometry.

Kyoto Encyclopedia of Genes and Genomes (KEGG) enrichment analysis was performed to decipher the biological functions of the identified DMP-associated genes. Consistent with the fact that withering affects the formation of flavor compounds in tea, the enriched KEGG pathways among the m^6^A-labeled genes were associated with genetic information processing (Figure 2A). More than half of the DMP-associated genes were assigned to the ribosome, RNA transport, and spliceosome pathways. Moreover, several DMP-associated genes were associated with three metabolism-related pathways. Among these pathways, the rich factor was highest for the terpenoid backbone biosynthesis pathway, which is closely related to the formation of the oolong tea aroma. We focused on the ribosome, RNA transport, spliceosome, and terpenoid backbone biosynthesis pathways in the subsequent analyses.

### Negative correlation between the m^6^A abundances and the expression levels of most DMP-associated genes under solar-withering conditions

**Supplementary Figure S8:**
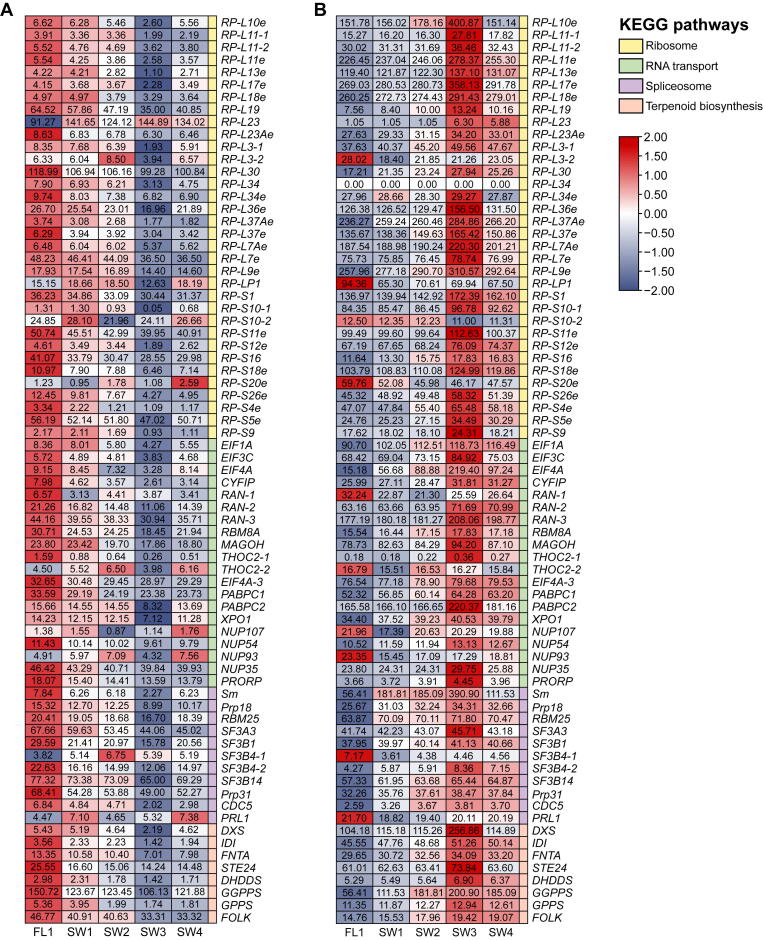
**m^6^A abundance and expression levels of DMP-associated genes under solar-withering with different shading rates based on RNA methylome and transcriptome datasets A.** m^6^A abundance of DMP-associated genes under solar-withering with different shading rates. **B.** Expression levels of DMP-associated genes under solar-withering with different shading rates. The m^6^A abundance and expression levels of DMP-associated genes were normalized using the *z*-score formula, respectively. FPKM, fragments per kilobase per million mapped reads; KEGG, kyoto encyclopedia of genes and genomes; m^6^A, *N*^6^-methyladenosine; FL, fresh leaves; SW1, solar-withering with a high shading rate; SW2, solar-withering with a middle shading rate; SW3, solar-withering with a low shading rate; SW4, solar-withering with natural sunlight.

The m^6^A-seq analysis showed that the m^6^A abundances and the expression levels of several core genes involved in the four aforementioned KEGG pathways were significantly altered during the solar-withering process (Figure S8A and B). In the ribosome pathway, the m^6^A abundances of 34 ribosomal protein-encoding (RP) genes were affected by the solar-withering treatment (Table S4). These RP genes are indispensable for ribosome formation and protein biosynthesis and have important roles in the transcription–translation process. We also determined that 20 and 16 DMP-associated genes are involved in RNA transport and spliceosome pathways, respectively (Tables S5 and S6). RNA transport is functionally linked to several steps in the RNA processing stage, including RNA splicing, 3′-end formation, and transcription–translation processes, which are critical for various biological functions [37]. AS is a pivotal post-transcriptional event that diversifies the generated transcripts and the corresponding proteins, making it a critical molecular mechanism that regulates transcriptional abundance in the plant kingdom. Interestingly, significant changes in the m^6^A levels were detected for eight terpenoid biosynthesis-related genes in response to the solar-withering treatment (Table S7).

**Supplementary Figure S9:**
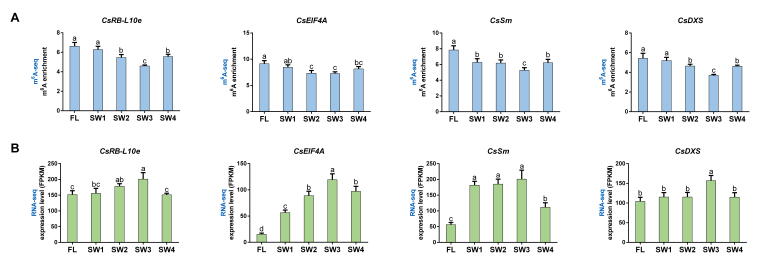
**m^6^A abundance and expression levels of *CsRB-L10e, CsEIF4A, CsSm*, and *CsDXS* under solar-withering with different shading rates based on RNA methylome and transcriptome datasets A.** m^6^A abundance of *CsRB-L10e, CsEIF4A, CsSm,* and *CsDXS* under solar-withering with different shading rates. **B.** Expression levels of *CsRB-L10e, CsEIF4A, CsSm*, and *CsDXS* under solar-withering with different shading rates. Data are presented as mean ± standard deviation. Different lowercase letters over the error bars indicate significantly different groups via one-way analysis of variance and Tukey’s *post hoc* test (*P* < 0.05). m^6^A, *N*^6^-methyladenosine; FPKM, fragments per kilobase per million mapped reads; FL, fresh leaves; SW1, solar-withering with a high shading rate; SW2, solar-withering with a middle shading rate; SW3, solar-withering with a low shading rate; SW4, solar-withering with natural sunlight.

For each of the four aforementioned pathways, we selected one DMP-associated gene in which there was a dynamic change in the m^6^A level following the solar-withering treatments with different shading rates. The four genes encode the large subunit ribosomal protein L10e (RB-L10e), eukaryotic translation initiation factor 4A (EIF4A), core spliceosomal Sm protein (Sm), and 1-deoxy-D-xylulose-5-phosphate synthase (DXS), respectively. The m^6^A peaks in their mRNA sequences were mainly distributed in the 3′ UTR and around the stop codon (Figure 2B). Our m^6^A-seq datasets revealed significant changes in the m^6^A levels of these four genes (Figure S9A). We then performed an m^6^A-immunoprecipitation-quantitative polymerase chain reaction (m^6^A-IP-qPCR) analysis to further validate the m^6^A levels of the four aforementioned genes. As expected, the m^6^A levels in *CsRB-L10e*, *CsEIF4A*, *CsSm*, and *CsDXS* decreased sharply from FLs to the SW3 leaves, but increased from the SW3 leaves to the SW4 leaves (Figure 2C). The expression levels of these four genes increased from FLs to the SW3 leaves, but then decreased in the SW4 leaves, as revealed by both transcriptome datasets (Figure S9B) and the quantitative real-time polymerase chain reaction (qRT-PCR) data (Figure 2D). These results indicate that the m^6^A modifications in these mRNAs were negatively correlated with their corresponding expression levels, which is in agreement with the findings of a previous study that demonstrated that mRNAs with low m^6^A levels tend to be highly expressed in tomato [11]. We detected obvious changes in the contents of seven monoterpenoids and one apocarotenoid using gas chromatography–mass spectrometry (GC–MS) (Figure 2E). Specifically, the abundances of all eight volatiles increased markedly from FLs to the SW3 leaves before decreasing significantly in the SW4 leaves. We also determined that the accumulation of eight volatile terpenoids was positively correlated with the *CsDXS* expression level, but negatively correlated with the m^6^A level of *CsDXS* and the overall m^6^A level under solar-withering conditions.

### Expression profiles of m^6^A regulatory genes in response to solar-withering

**Figure 3:**
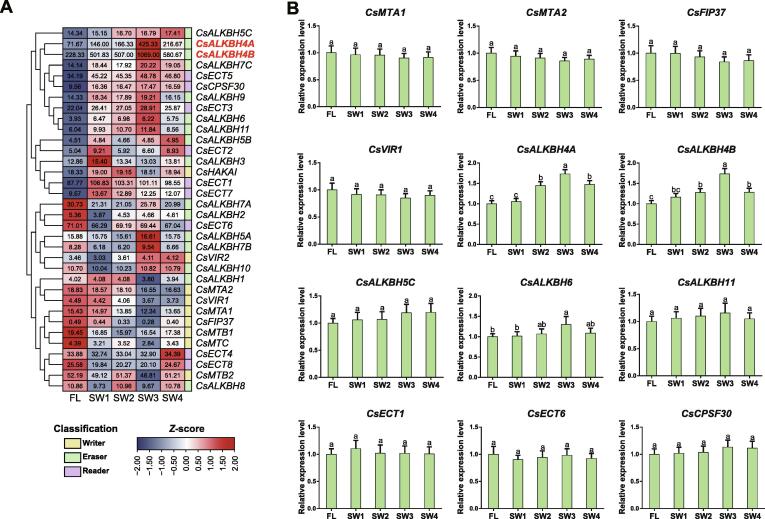
**Expression profiles of m^6^A regulatory genes under solar-withering with different shading rates** **A.** The heatmap of m^6^A regulatory genes under solar-withering with different shading rates based on the transcriptome datasets. The expression values of m^6^A regulatory genes were normalized using *z*-score formula. The number in the box indicates the original FPKM value. **B.** Expression patterns of four m^6^A writer genes, five m^6^A eraser genes, and three m^6^A reader genes under solar-withering with different shading rates determined by qRT-PCR. Data are presented as mean ± SD. Different lowercase letters over the error bars indicate significantly different groups via one-way ANOVA and Tukey’s *post hoc* test (*P* < 0.05). FPKM, fragments per kilobase per million mapped reads.

The effects of RNA methylation are tightly associated with the transcript levels of RNA methyltransferase, demethylase, and reader genes [38]. Thus, we speculated that the m^6^A levels of these mRNAs are coordinately governed by m^6^A regulatory genes. Based on our recent report [14], 34 m^6^A regulatory genes were identified in the tea reference genome. We initially analyzed the expression profiles of m^6^A regulatory genes under solar-withering conditions (Figure 3A). The m^6^A regulatory genes with |FC| ≥ 2 and *P* < 0.05 were considered to be differentially expressed between samples. The transcript levels of all m^6^A writer and reader genes were not obviously altered by the solar-withering conditions. Among the 16 m^6^A eraser genes, only *CsALKBH4A* and *CsALKBH4B* had significant changes in their transcript levels after the solar-withering treatments. We also noticed that *CsALKBH6* transcription was significantly induced only following the SW3 treatment. These results indicate that dynamic changes in m^6^A modifications may be mainly controlled by m^6^A eraser genes, with *CsALKBH6* playing a particularly important role in the regulation of m^6^A levels in response to SW3. Next, a qRT-PCR analysis confirmed the expression patterns of m^6^A regulatory genes revealed by the transcriptome data, and demonstrated that the *CsALKBH4A* and *CsALKBH4B* expression levels increased continuously from FLs to the SW3 leaves, but then clearly decreased in the SW4 leaves (Figure 3B). This trend was in accordance with the expression patterns of *CsRB-L10e*, *CsEIF4A*, *CsSm*, and *CsDXS*, but was inversely correlated with the m^6^A abundances of these four genes and the global m^6^A level under solar-withering conditions. Moreover, the *CsALKBH6* expression level increased significantly only following the SW3 treatment, whereas the expression of the m^6^A writer and reader genes was unaffected by the solar-withering treatments. These observations imply that the m^6^A eraser-mediated removal of m^6^A marks on mRNAs is closely linked with variations in m^6^A levels and the expression of DMP-associated genes.

### CsALKBH4-mediated RNA demethylation may activate terpenoid biosynthesis

**Figure 4:**
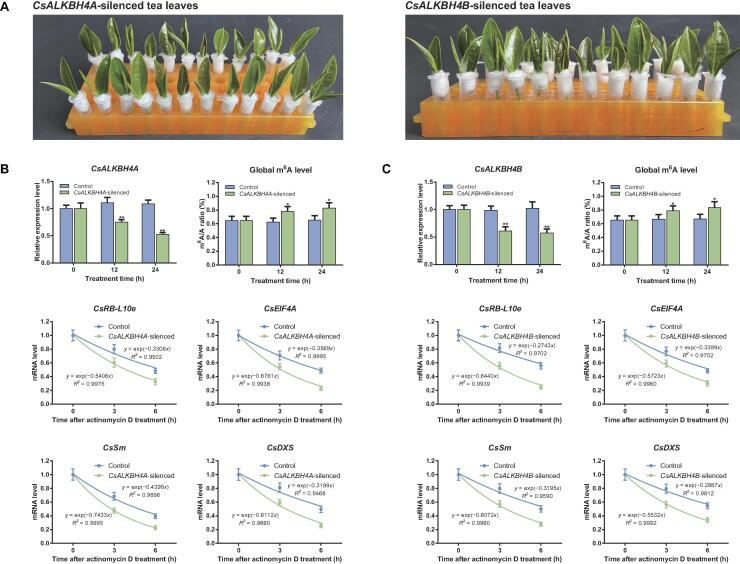
**CsALKBH4-mediated RNA demethylation contributes to the accumulation of volatile terpenoids by removing the m^6^A marks and enhancing the stability of corresponding mRNAs** **A.** Diagram of *CsALKBH4A-* and *CsALKBH4B*-silenced assay in tea leaves via an siRNA-mediated gene silencing strategy. The freshly detached tea bud and first leaf from naturally grown tea plants were incubated in 1.5-ml microcentrifuge tubes that contained 1 ml of 20 μM siRNA or siRNA-NC solution. After incubation for 12 h and 24 h, the tea bud and first leaf were harvested and then used for qRT-PCR and metabolite detection. **B.** Gene expression, global m^6^A level, and mRNA stability in *CsALKBH4A-*silenced tea leaves. **C.** Gene expression, global m^6^A level, and mRNA stability in *CsALKBH4B-*silenced tea leaves. To evaluate the mRNA stability, the leaf discs were collected from gene-silenced tea leaves and incubated in sterile water that contained 10 μg/ml actinomycin D solution. Tea leaves incubated in sterile water were used as controls. Total RNA was isolated from the leaves sampled at 6 h and 12 h, respectively. The mRNA levels of *CsRB-L10e*, *CsEIF4A*, *CsSm*, and *CsDXS* were examined by qRT-PCR. Data are presented as mean ± SD. The differences among various groups were assessed by conducting a one-way ANOVA and Tukey’s *post hoc* test. *, *P* < 0.05; **, *P* < 0.01. siRNA-NC, negative control siRNA.

**Figure 5:**
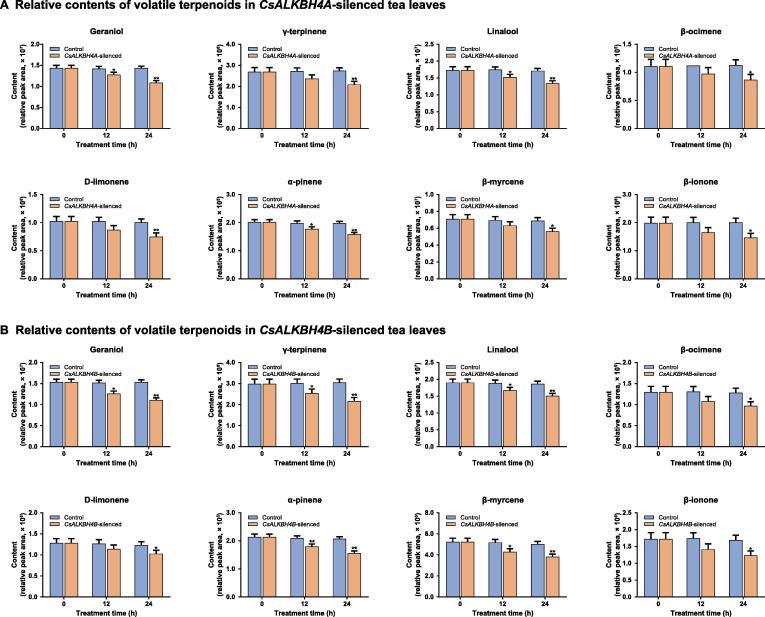
**Relative contents of volatile terpenoids in*****CsALKBH4A*****/*****CsALKBH4B******-*silenced tea leaves determined by GC–MS** **A.** Relative contents of volatile terpenoids in *CsALKBH4A-*silenced tea leaves. **B.** Relative contents of volatile terpenoids in *CsALKBH4B-*silenced tea leaves. Data are presented as mean ± SD. The differences among various groups were assessed by conducting a one-way ANOVA and Tukey’s *post hoc* test. *, *P* < 0.05; **, *P* < 0.01.

**Supplementary Figure S10:**
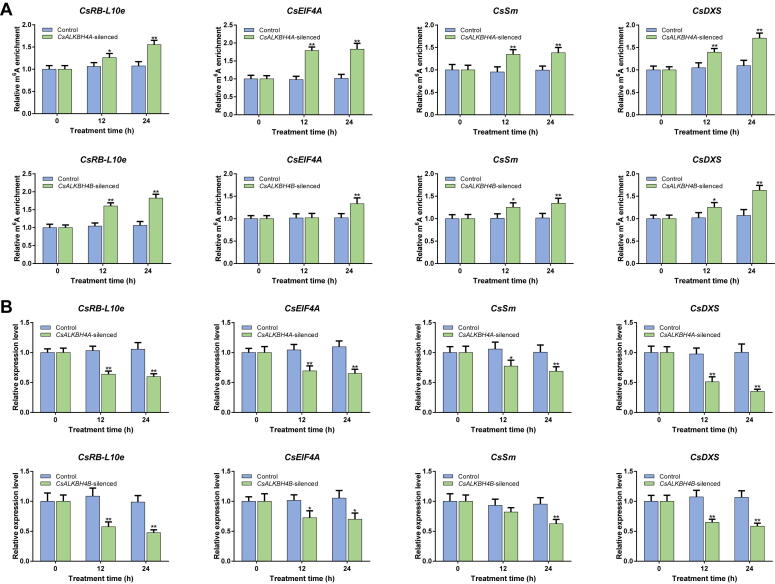
**Relative m^6^A enrichment and relative expression levels of *CsRB-L10e, CsEIF4A, CsSm*, and *CsDXS* in *CsALKBH4A-* or *CsALKBH4B*-silenced leaves A.** Relative m^6^A enrichment of *CsRB-L10e, CsEIF4A, CsSm*, and *CsDXS* in *CsALKBH4A*- or *CsALKBH4B*-silenced leaves determined by m^6^A-IP-qPCR. **B.** Relative expression levels of *CsRB-L10e, CsEIF4A, CsSm,* and *CsDXS* in *CsALKBH4A*- or *CsALKBH4B*-silenced leaves determined by qRT-PCR. Data are presented as mean ± standard deviation. The differences among various groups were assessed by conducting a one-way analysis of variance and Tukey’s *post hoc* test. *, *P* < 0.05; **, *P* < 0.01; m^6^A, *N*^6^-methyladenosine; m^6^A-IP-qPCR, m^6^A-immunoprecipitation-quantitative polymerase chain reaction; qRT-PCR, quantitative real-time polymerase chain reaction.

To further functionally characterize *CsALKBH4A* and *CsALKBH4B* during the solar-withering process, we transiently suppressed the expression of these two genes via an siRNA-mediated gene silencing strategy (Figure 4A). As anticipated, the expression of both *CsALKBH4A* and *CsALKBH4B* was significantly down-regulated in the gene-silenced leaves, whereas the transcription of these two genes was not substantially altered by treatments with the corresponding negative control (NC) (Figure 4B and C). The inhibited expression of *CsALKBH4A* and *CsALKBH4B* led to a marked increase in the overall m^6^A level in the gene-silenced tea leaves (Figure 4B and C), which occurred concomitantly with an obvious increase in the m^6^A levels of *CsRB-L10e*, *CsEIF4A*, *CsSm*, and *CsDXS* (Figure S10A) (compared with the respective NC). Conversely, the *CsRB-L10e*, *CsEIF4A*, *CsSm*, and *CsDXS* mRNA levels were markedly lower in the gene-silenced leaves than in the NC-treated leaves (Figure S10B). Accordingly, the diminished expression of these four genes may be mainly attributed to the suppression of CsALKBH4-mediated RNA demethylation. To further investigate how m^6^A demethylation influences the transcription of *CsRB-L10e*, *CsEIF4A*, *CsSm*, and *CsDXS*, we explored whether CsALKBH4-mediated RNA demethylation modulates the stability of the transcripts of these four genes by monitoring mRNA decay rates after an actinomycin D treatment (Figure 4B and C). We observed that the *CsRB*-*L10e*, *CsEIF4A*, *CsSm*, and *CsDXS* mRNAs degraded more rapidly in the *CsALKBH4A*- and *CsALKBH4B*-silenced leaves than in the NC-treated leaves. Notably, for *CsRB*-*L10e*, *CsEIF4A*, *CsSm*, and *CsDXS*, their mRNA decay rates were negatively correlated with their expression levels and positively correlated with their m^6^A levels. These results suggest that excessive m^6^A modifications in the *CsRB*-*L10e*, *CsEIF4A*, *CsSm*, and *CsDXS* mRNAs have a destabilizing effect, which results in a clear decrease in transcript levels. To assess the effects of RNA demethylation on the accumulation of volatile terpenoids, the terpenoid contents in *CsALKBH4A*- and *CsALKBH4B*-silenced leaves were measured. The transcriptional repression of these two genes resulted in a substantial decrease in the contents of the eight volatile terpenoids (Figure 5A and B), suggesting that CsALKBH4-mediated RNA demethylation may directly activate terpenoid biosynthesis by removing m^6^A marks and enhancing the stability of the corresponding mRNAs.

### RNA demethylation modulates AS events by influencing m^6^A modifications and the expression of spliceosome-related genes

The spliceosome pathway was one of the main enriched KEGG pathways among the DMP-associated genes. The m^6^A modifications of the core genes *Sm*, *Prp18* (encoding pre-mRNA-splicing factor 18), and *Prp31* (encoding pre-mRNA-splicing factor 31) in the spliceosome pathway were significantly altered by the solar-withering treatment, which was consistent with the obvious expression changes detected for these genes. Thus, we hypothesized that RNA demethylation might contribute to the regulation of AS events by controlling the m^6^A levels and the expression levels of spliceosome-related genes. To test this hypothesis, we first investigated the AS events during the solar-withering treatments with different shading rates. A total of 18,188 AS events were detected in 13,606 genes in five tea samples. There were substantially more AS events in the solar-withered leaves than in FLs, suggesting that solar-withering conditions may influence the occurrence of AS events (Table S8). Within a certain shading rate range (*i.e.*, SW1–SW3), the frequency of AS events was closely correlated with the decreases in the shading rate. In contrast, solar-withering without shading substantially decreased the number of AS events. In the examined samples, the predominant AS events resulted in retained introns. This observation is in accordance with the results of earlier investigations on maize [39], cotton [40], and tea [41]. We next comprehensively identified the differentially expressed alternative splicing genes (DAGs) among the solar-withering treatments with different shading rates. There were more DAGs in the FL *vs.* SW3 comparison than in the FL *vs.* SW1 and FL *vs.* SW2 comparisons, implying that increasing the light intensity of the solar-withering treatment may promote the differential expression of AS genes (Table S9). However, there were fewer DAGs in the FL *vs.* SW4 comparison than in the FL *vs.* SW3 comparison. These findings indicate that the shading rate during the solar-withering treatment affects the number of AS events, possibly by modulating the expression of AS genes.

### Association between flavonoid, catechin, and theaflavin contents and the AS gene transcript levels under solar-withering conditions

**Figure 6:**
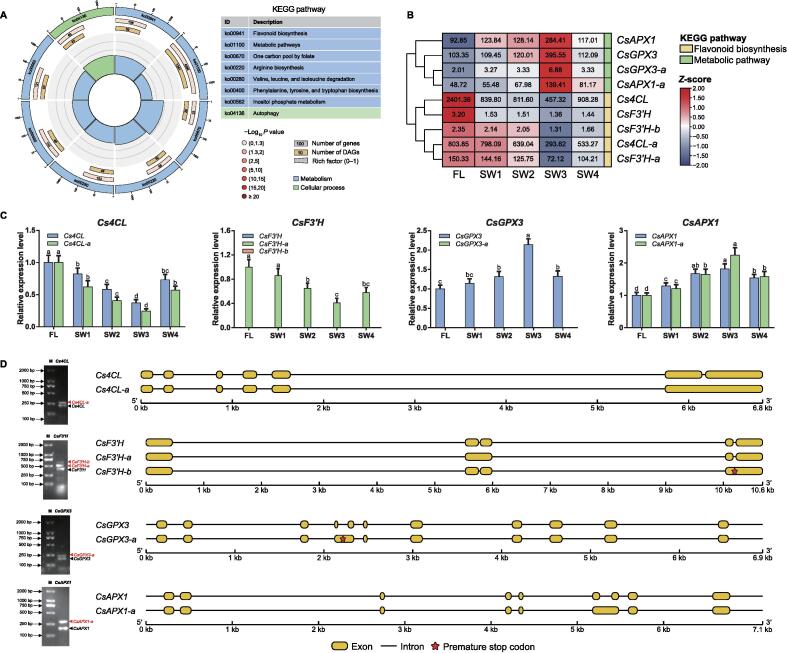
**AS-mediated regulatory mechanism influences the accumulation of flavor-related metabolites** **A.** KEGG enrichment analysis of DAGs. From the outside to the inside, the first circle indicates enriched KEGG pathways and the number of genes corresponds to the outer circle. The second circle indicates the number of genes in the genome background and the *P* value for the enrichment of the genes in the specified KEGG pathway. The third circle indicates the number of DAGs. The fourth circle indicates the enrichment factor of each KEGG pathway. **B.** The heatmap of DAGs and their AS transcripts under solar-withering with different shading rates based on the transcriptome datasets. The expression values of DAGs and their AS transcripts were normalized using the *z*-score formula. The number in the box indicates the original FPKM value. **C.** Expression patterns of *Cs4CL*, *CsF3′H*, *CsGPX3*, and *CsAPX1* as well as their AS transcripts under solar-withering with different shading rates determined by qRT-PCR. Data are presented as mean ± SD. Different lowercase letters over the error bars indicate significantly different groups via one-way ANOVA and Tukey’s *post hoc* test (*P* < 0.05). **D.** RT-PCR validation and gene structure of the full-length transcripts and AS transcripts. M indicates the lane with DNA size markers. The full-length transcripts and AS transcripts on the gel images are denoted with black and red triangles, respectively. AS, alternative splicing; DAG, differentially expressed alternative splicing gene; RT-PCR, reverse transcription-polymerase chain reaction.

**Supplementary Figure S11:**
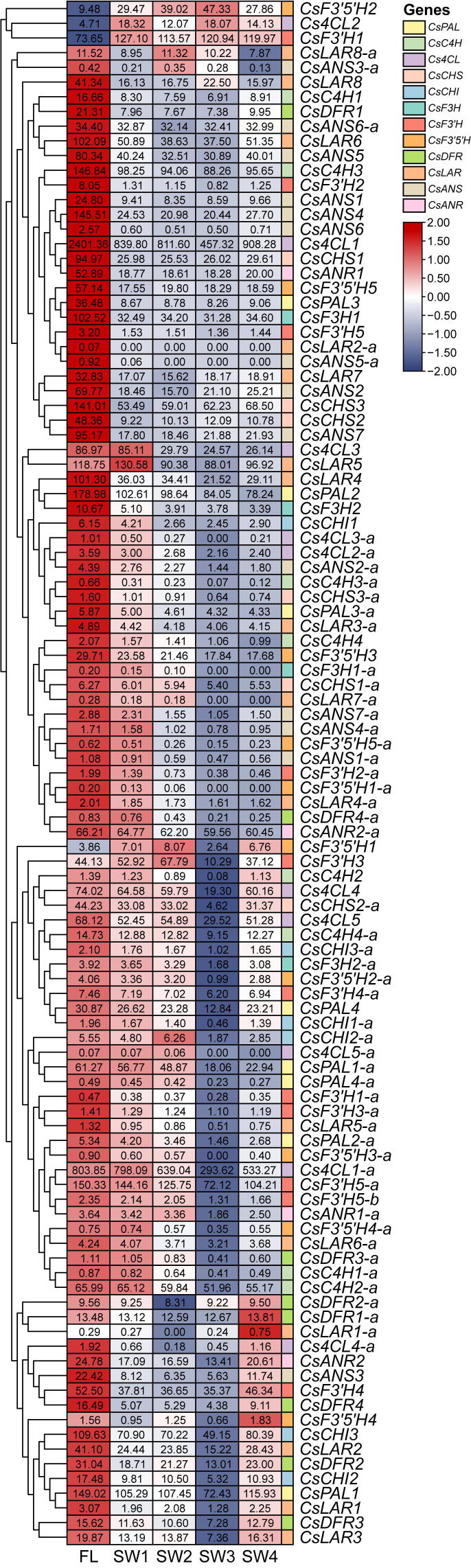
**Expression levels of DAGs and their AS transcripts involved in flavonoid biosynthesis pathway under solar-withering with different shading rates based on transcriptome datasets** Expression values of DAGs and their AS transcripts were normalized using the *z*-score formula. DAGs, differentially expressed alternative splicing genes; AS, alternative splicing; FL, fresh leaves; SW1, solar-withering with a high shading rate; SW2, solar-withering with a middle shading rate; SW3, solar-withering with a low shading rate; SW4, solar-withering with natural sunlight.

**Supplementary Figure S12:**
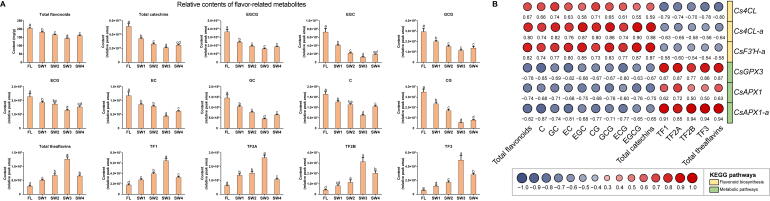
**Relative contents of flavor-related metabolites and correlation analyses of full-length transcripts, AS transcripts, and flavor metabolites A.** Relative contents of flavonoids, catechins, and theaflavins under solar-withering with different shading rates determined by LC-MS. Data are presented as mean ± standard deviation. Different lowercase letters over the error bars indicate significantly different groups via one-way analysis of variance and Tukey’s *post hoc* test (*P* < 0.05). **B.** Correlation analyses of full-length transcripts, AS transcripts, and flavor metabolites. The correlation analyses were performed based on Pearson’s correlation coefficient. The number below the circle indicates the corresponding Pearson’s correlation coefficient. A correlation coefficient greater than 0 indicates a positive correlation, while a correlation coefficient less than 0 indicates a negative correlation. LC-MS, liquid chromatography-mass spectrometry; FL, fresh leaves; SW1, solar-withering with a high shading rate; SW2, solar-withering with a middle shading rate; SW3, solar-withering with a low shading rate; SW4, solar-withering with natural sunlight; C, catechin; CG, catechin gallate; EC, epicatechin; ECG, epicatechin gallate; EGC, epigallocatechin; EGCG, epigallocatechin gallate; GC, gallocatechin; GCG, gallocatechin gallate; TF1, theaflavin; TF2A, theaflavin-3-gallate; TF2B, theaflavin-3′-gallate; TF3, theaflavin-3,3′-digallate; KEGG, kyoto encyclopedia of genes and genomes.

To further explore the putative effects of DAGs under solar-withering conditions, we performed a KEGG analysis of all identified DAGs. Metabolic pathways were the most enriched pathways among the DAGs, followed by the flavonoid biosynthesis pathway (Figure 6A). Therefore, structural genes involved in flavonoid biosynthesis may be affected by the m^6^A-mediated AS regulatory mechanism (Figure S11; Table S10). We selected *4CL* (encoding 4-coumarate CoA ligase) and *F3′H* (encoding flavonoid 3′-hydroxylase) as two flavonoid biosynthesis-related DAGs for further analyses. AS events generated two and three splicing variants for *4CL* and *F3′H*, respectively. On the basis of the fragments per kilobase per million mapped reads (FPKM) values obtained from the transcriptome datasets, the expression profiles of these AS transcripts under solar-withering conditions were analyzed (Figure 6B). The expression of the full-length *Cs4CL* transcript decreased substantially from FLs to the SW3 leaves, but then obviously increased in the SW4 leaves. The expression profile of the AS transcript *Cs4CL-a* was similar to that of the full-length transcript after the solar-withering treatment, whereas the *Cs4CL-a* transcript abundance was lower than that of *Cs4CL* following the same solar-withering treatment. Solar-withering also strongly inhibited the transcription of *CsF3′H-a*. Because of the high cycle threshold value > 35 for *CsF3′H* and *CsF3′H-b* in the qRT-PCR assay (Table S11), the expression levels for these two transcripts were extremely low in FLs and the solar-withered leaves. Hence, *CsF3′H-a* may be the predominant transcript involved in flavonoid biosynthesis. The qRT-PCR analysis confirmed the transcriptome datasets were reliable (Figure 6C). To identify the AS transcripts related to the biosynthesis of flavonoids and catechins during solar-withering with different shading rates, the flavonoid and catechin contents in all five samples were compared (Figure S12A). There was a sharp decrease in the total flavonoid content from FLs to the SW3 leaves, but there was a distinct rebound from SW3 to SW4.

Excessive and insufficient amounts of catechins have detrimental effects on tea flavor and the health benefits of tea. In this study, we examined whether the solar-withering process increases the palatability of oolong tea, while also decreasing the health benefits of tea. Intriguingly, AS events were detected for *APX1* (encoding ascorbate peroxidase 1) and *GPX3* (encoding glutathione peroxidase 3), which are involved in metabolic pathways. These two genes are reportedly responsible for the conversion of catechins into theaflavins [42], [43]. We hypothesized that the solar-withering treatments strongly inhibited catechin biosynthesis, resulting in an obvious increase in the theaflavin content. To assess this hypothesis, the total theaflavin content and the contents of four theaflavin components were measured using liquid chromatography–mass spectrometry (LC–MS). As expected, the solar-withering treatments caused the individual theaflavin contents and the total theaflavin content to increase substantially (Figure S12A). Additionally, the theaflavin contents were inversely correlated with the catechin contents under solar-withering conditions. Next, we observed that the *CsGPX3*, *CsAPX1*, and *CsAPX1*-*a* expression levels increased dramatically from FLs to the SW3 leaves, but then clearly decreased in the SW4 leaves. One AS transcript (*CsGPX3*-*a*) was unaffected by the solar-withering treatments. We then detected a premature stop codon (PTC) in the *CsGPX3*-*a* mRNA (Figure 6D).

**Supplementary Figure S13:**
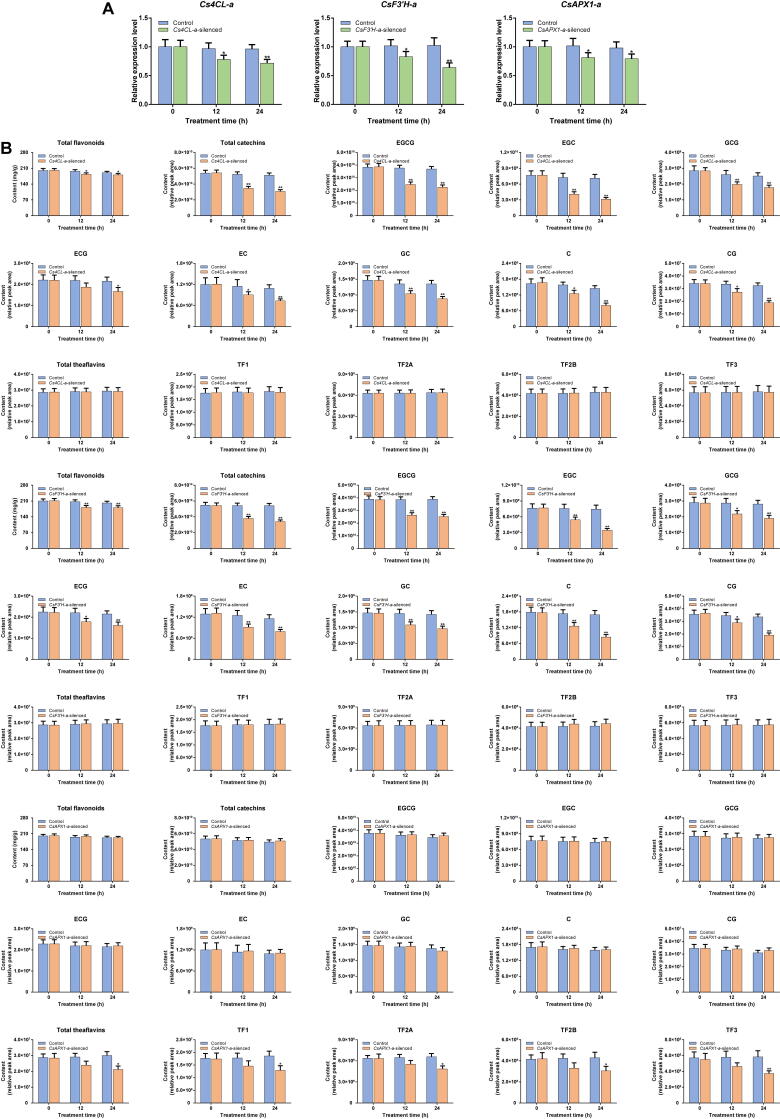
**Relative expression levels of *Cs4CL-a, CsF3'H-a,* and *CsAPX1-a*, as well as the contents of flavor metabolites in AS transcript-silenced leaves A.** Relative expression levels of *Cs4CL-a, CsF3'H-a*, and *CsAPX1-a* in AS transcript-silenced leaves determined by qRT-PCR. **B.** The contents of flavor metabolites in AS transcript-silenced leaves were determined by LC-MS. Data are presented as mean ± standard deviation. The differences among various groups were assessed by conducting a one-way analysis of variance and Tukey’s *post hoc* test. *, *P* < 0.05; **, *P* < 0.01; C, catechin; CG, catechin gallate; EC, epicatechin; ECG, epicatechin gallate; EGC, epigallocatechin; EGCG, epigallocatechin gallate; GC, gallocatechin; GCG, gallocatechin gallate; TF1, theaflavin; TF2A, theaflavin-3-gallate; TF2B, theaflavin-3′-gallate; TF3, theaflavin-3,3′-digallate.

To further elucidate the possible effects of AS transcripts on the accumulation of flavonoids, catechins, and theaflavins, the relationships between the abundances of flavonoid-related AS transcripts and metabolites were evaluated via Pearson’s correlation analysis (Figure S12B). Three transcripts (*CsF3′H*, *CsF3′H-b*, and *CsGPX3*-*a*) were excluded from this analysis because they were not expressed in FLs and the solar-withered leaves. The expression levels of the full-length *Cs4CL* transcript and the *Cs4CL*-*a* AS transcript were positively correlated with the total flavonoid, total catechin, and eight individual catechin contents. Positive correlations were also detected between the expression level of the *CsF3′H*-*a* AS transcript and the abundances of the aforementioned metabolites. Moreover, we observed that the accumulation of four individual theaflavin components and the total theaflavin content were positively correlated with the expression of *CsGPX3*, *CsAPX1*, and *CsAPX1*-*a*. We also examined the effects of AS transcripts (*Cs4CL-a*, *CsF3′H-a*, and *CsAPX1-a*) on the accumulation of the associated metabolites (Figure S13A). As expected, the *Cs4CL-a*, *CsF3′H-a*, and *CsAPX1-a* transcript levels were considerably down-regulated in the gene-silenced leaves, whereas they were not obviously altered by the corresponding NC treatments. Next, the flavonoid, catechin, and theaflavin contents in the gene-silenced leaves were analyzed (Figure S13B). The total flavonoid and total catechin contents as well as the accumulation of eight individual catechins decreased sharply following the silencing of *Cs4CL-a* or *CsF3′H-a*, which was in contrast to the lack of any significant changes to the total theaflavin content and the abundances of four individual theaflavins. The transcriptional repression of *CsAPX1-a* did not affect the flavonoid and catechin contents in the gene-silenced leaves, but it inhibited the accumulation of theaflavins. These observations imply that some AS transcripts are likely critical for the accumulation of flavonoids, catechins, and theaflavins.

## Discussion

### Dynamic changes in global m^6^A level in tea leaves are mainly controlled by m^6^A erasers under solar-withering conditions

In the past few decades, several studies on the environmental stresses during tea manufacturing (*e.g.*, withering stage) have clarified the molecular basis of specific metabolic activities related to tea flavor formation [15], [17]. Recent research confirmed epigenetic modifications, such as DNA methylation and histone modifications, influence the production of tea flavor-related substances [28], [29]. However, it remains unclear whether RNA methylation (epitranscriptome-level changes) also regulates flavor-related metabolic pathways and tea flavor formation.

In the present study, we observed that m^6^A modifications are widely distributed among mRNAs in tea plants, with dynamic changes in the m^6^A levels induced by solar-withering treatments. More specifically, in response to solar-withering conditions, the overall m^6^A level decreased dramatically. From FLs to the SW3 leaves, the overall m^6^A level gradually decreased, but the m^6^A level increased in the SW4 leaves. Thus, solar-withering treatments significantly decreased the m^6^A levels. Moreover, there was a positive correlation between the shading rate and the global m^6^A abundance within a certain range. According to earlier investigations [44], [45], the overall DNA methylation level is mediated by DNA methyltransferases and demethylases. In addition, the corresponding RNA methyltransferases and demethylases have been identified in tea plants [14]. Therefore, we speculated that the overall m^6^A level may be controlled by RNA methyltransferases and demethylases under solar-withering conditions. To evaluate this hypothesis, we comprehensively examined the expression profiles of m^6^A regulatory genes following solar-withering treatments. Notably, obvious transcript-level changes were detected in two m^6^A eraser genes (*CsALKBH4A* and *CsALKBH4B*) during the solar-withering treatments with different shading rates, whereas the expression levels of other m^6^A eraser and reader genes were only slightly affected by solar-withering. These findings are consistent with the reported phenomena in *A. thaliana* [46] and tomato [11]. Similarly, during the solar-withering stage, tea leaves are subjected to multiple environmental stresses, including UV radiation [15]. Thus, our results imply that the removal of m^6^A marks, rather than the addition and decoding of these marks, may be vital for tea plant responses to multiple stresses during the tea-withering stage. The inclusion of sunshade nets in solar-withering treatments prevents the excessive UV irradiation of tea leaves, thereby minimizing the damages to the living tea leaves. The *CsALKBH4A* and *CsALKBH4B* transcription levels increased continuously as the shading rate increased (from FLs to the SW3 leaves). Furthermore, the global m^6^A level was negatively correlated with the expression of these two m^6^A eraser genes. Additionally, the overall m^6^A level increased in the *CsALKBH4*-silenced leaves. These observations suggest that m^6^A demethylation is primarily responsible for the decrease in the global m^6^A level from FLs to the SW3 leaves. However, tea leaves were exposed to high UV doses during the solar-withering without the sunshade net (SW4). Exposures to high UV doses may lead to irreversible damages to the osmotic regulatory activities of plasma membranes [47], [48], [49], ultimately leading to ruptured cells and further disruptions to the normal expression of the nuclear-localized genes *CsALKBH4A* and *CsALKBH4B* [14]. In the current study, low UV doses induced *CsALKBH4* expression within a certain range during the solar-withering treatments with the sunshade net, whereas high UV doses strongly inhibited *CsALKBH4* expression when the solar-withering treatment was completed without a sunshade net. The down-regulated expression of *CsALKBH4* in response to the SW4 treatment may have impaired the ability of m^6^A erasers to remove m^6^A modifications, which may help to explain the observed increase in the overall m^6^A level from SW3 to SW4. Collectively, these results indicate that the dynamic changes in global m^6^A level in tea leaves undergoing solar-withering treatments with different shading rates are mainly controlled by m^6^A erasers. In addition, CsALKBH4-mediated RNA demethylation likely affects critical processes during the tea-withering stage.

### RNA demethylation directly contributes to the accumulation of volatile terpenoids and the formation of tea aromas

Environmental stresses can considerably affect the accumulation of specialized metabolites in tea leaves, thereby affecting tea quality [50]. During postharvest processing, tea leaves are exposed to various environmental stresses, which induce obvious alterations to many flavor-related compounds, leading to the production of teas with unique flavors [17]. Although the effects of specific metabolites on tea flavor formation have been investigated at the transcriptional, translational, and metabolic levels, the functional roles of epigenetic modifications, especially RNA methylation, and the regulatory mechanisms underlying the m^6^A-mediated flavor formation in the tea-withering stage remain unclear.

In the current study, an integrated RNA methylome and transcriptome analysis revealed that the variations in the number of DMPs in response to solar-withering with different shading rates were in accordance with the changes in the total m^6^A level. The number of DMPs decreased between the FL *vs.* SW1 and FL *vs.* SW3 comparisons, but it increased in the FL *vs.* SW4 comparison. This increase may be associated with the inhibition of *CsALKBH4* expression due to high UV doses during solar-withering without a sunshade net. The down-regulated expression of *CsALKBH4* in the SW4 leaves hindered RNA demethylation, thereby increasing the differences in the m^6^A levels between the SW4 leaves and the leaves that underwent the other treatments and finally leading to an increase in the number of DMPs. A total of 1137 genes with DMPs under solar-withering conditions were functionally characterized. The KEGG pathway analysis showed that the identified DMP-associated genes were mainly associated with the terpenoid biosynthesis pathway. Volatile terpenoids are major quality-related compounds in oolong tea. Specifically, they significantly influence the formation of floral and honey-like aromas in high-quality oolong tea because of their low odor thresholds [15]. Moreover, several lines of evidence suggest that environmental stresses can alter the m^6^A levels of transcripts [12], [51]. In our study, the expression levels of seven terpenoid biosynthesis-related genes increased significantly under solar-withering conditions, which may be related to the observed considerable accumulation of volatile terpenoids. The positive correlations between the expression levels of these structural genes related to terpenoid biosynthesis and terpenoid contents imply that multiple stresses induced by solar-withering promote terpenoid accumulation by up-regulating the expression of terpenoid biosynthesis-related genes. This is in accordance with the results of another study [18]. In addition, the m^6^A levels in these terpenoid biosynthesis-related genes were negatively correlated with the corresponding expression levels. Similarly, an earlier investigation has demonstrated that the expression of genes with low m^6^A levels tends to be up-regulated [52]. These results suggest that the expression levels of m^6^A-containing genes may be governed by RNA methylation. According to recent reports, knocking out m^6^A writer genes can dramatically decrease the m^6^A levels of m^6^A-modified genes, which results in marked increases in gene expression levels [6], [53]. In an earlier study, the overaccumulation of m^6^A marks on mRNAs adversely affected gene expression in the *AtALKBH10B* mutant line, suggesting that m^6^A modifications may negatively affect mRNA expression [54]. In our study, the suppression of *CsALKBH4A* and *CsALKBH4B* expression resulted in a significant decrease in the stability of *CsDXS* mRNA and the transcripts of three DMP-associated genes. Furthermore, the abundances of eight volatile terpenoids decreased in the *CsALKBH4A*- or *CsALKBH4B*-silenced leaves. These findings along with the CsALKBH4-mediated RNA demethylation mentioned above suggest that RNA demethylation may regulate the overall m^6^A level by modulating the expression of m^6^A eraser genes, while also stabilizing the mRNAs of DMP-associated genes, thereby increasing their expression. However, some reports indicate that mRNA abundance is positively correlated with m^6^A modifications [13], [55], which is inconsistent with our data. Therefore, the mechanism underlying the regulatory effects of m^6^A modifications on mRNAs in the solar-withering stage will need to be more thoroughly characterized. A recent study concluded that abiotic stress can alter the location of m^6^A peaks in transcripts [9]. The data generated in the present study showed that m^6^A peaks in *CsDXS* and three other DMP-associated genes were distributed within the 3′ UTR and around the stop codon under solar-withering conditions, indicating that solar-withering can affect the m^6^A levels in these DMP-associated genes, but it cannot induce the redistribution of m^6^A marks. Upon reviewing the reports that m^6^A modifications positively affect mRNA abundance, we noted that these m^6^A marks are mainly concentrated in the CDS region. This observation along with our other findings indicates that m^6^A modifications may have distinct regulatory effects on mRNA expression depending on their distribution in the transcript structure. Considered together, these findings suggest that CsALKBH4-mediated RNA demethylation promotes the expression of DMP-associated genes involved in terpenoid biosynthesis and the accumulation of aroma-related terpenoids by enhancing mRNA stability. Additionally, the shading rate was negatively correlated with the expression of terpenoid biosynthesis-related genes and terpenoid contents from FLs to the SW3 leaves, implying that within a certain range, moderate shading promotes terpenoid biosynthesis and the formation of a high-quality aroma in oolong tea. Inadequate shading during the SW4 treatment inhibited CsALKBH4-mediated RNA demethylation, with the detrimental effects on the expression of terpenoid biosynthesis-related genes ultimately leading to a significant decrease in the terpenoid content. This is consistent with the beneficial effects of moderate shading on flavor formation during the production of teas. Hence, solar-withering treatments of tea leaves may be optimized by controlling the shading rate, which is critical for enhancing tea aromas.

### RNA modifications indirectly affect the contents of flavonoid, catechin, and theaflavin by triggering the AS regulatory mechanism

The AS regulatory mechanism is a crucial component of plant responses to diverse environmental stimuli [56]. The generation of many AS transcripts leads to the expression of various proteins that alleviate the adverse effects of multiple stresses. Although many AS events related to secondary metabolism have been detected, the specific mechanisms regulating AS events associated with flavor-related genes and tea flavor formation during the tea manufacturing process are still unclear. Recent research found that m^6^A modifications have important regulatory effects on RNA splicing [38]. Likewise, we observed that CsALKBH4-mediated RNA demethylation influences *CsSm* mRNA abundance in the spliceosome pathway by enhancing its stability. According to previous research, *Sm* encodes a crucial spliceosome component [57] that interacts with several small nuclear RNAs (snRNAs) and then binds to a series of additional proteins to form small nuclear ribonucleoprotein particles. Within the spliceosome, snRNAs contribute to the catalysis and recognition of splice sites during pre-mRNA splicing. Intriguingly, both the frequency of AS events and the number of DAGs were closely associated with *CsSm* expression. Therefore, RNA demethylation might regulate AS events by modulating the m^6^A abundance and the expression of spliceosome-related genes.

To further investigate AS-mediated regulatory effects under solar-withering conditions, we performed a KEGG pathway enrichment analysis of all identified DAGs. Most of the DAGs were assigned to metabolic pathways and the flavonoid biosynthesis pathway. The subsequent analysis of the expression of four DAGs involved in these pathways revealed that the AS transcripts with the PTC (*CsF3′H*-*b* and *CsGPX3*-*a*) were expressed at almost undetectable levels under solar-withering conditions. This phenomenon may reflect the introduction of PTC into the gene structure, leading to the production of loss-of-function truncated proteins, which may be degraded via the nonsense-mediated decay pathway. This possibility is supported by the results of the analysis of the correlation between metabolite accumulation and the expression of AS transcripts, which revealed a lack of correlation between *CsF3′H*-*b* expression and the accumulation of flavonoids and catechins. Additionally, *CsGPX3*-*a* expression was only marginally correlated with the theaflavin level. Notably, the expression patterns of two non-PTC-type AS transcripts (*Cs4CL-a* and *CsF3′H-a*) were consistent with those of the corresponding full-length transcripts under solar-withering conditions. Meanwhile, *Cs4CL-a* and *CsF3′H-a* were more highly expressed than their full-length transcripts regardless of the shading rate during the solar-withering treatment. These findings imply that these two AS transcripts are the predominant transcripts in the flavonoid biosynthesis pathway. Similarly, *CsbHLH-2*, which is an AS transcript, is commonly formed in response to exposure to cold stress, during which it enhances stress tolerance by positively regulating certain signaling pathways [58]. Another AS transcript (*CsAPX1*-*a*) and its full-length transcript may participate in the coordinated regulation of theaflavin biosynthesis. The AS-mediated regulation of flavor metabolites was supported by our analysis of the correlation between AS transcripts and metabolites under solar-withering conditions, which revealed that *Cs4CL-a* and *CsF3′H-a* are positively correlated with the accumulation of flavonoids and catechins. A positive correlation was also detected between the AS transcript *CsAPX1*-*a* and the theaflavin content. We propose that the accumulation of flavonoids, catechins, and theaflavins is mediated by the canonical full-length transcripts as well as by particular AS transcripts, which may be important for the post-transcriptional regulation induced by solar-withering. Therefore, the down-regulated expression of *Cs4CL*, *Cs4CL*-*a*, and *CsF3′H*-*a* as well as the up-regulated expression of *CsGPX3*, *CsAPX1*, and *CsAPX1-a* in solar-withered leaves synergistically modulates flavonoid biosynthesis and flavonoid catabolism to promote the conversion of compounds associated with bitterness and astringency (*e.g.*, flavonoids and catechins) into theaflavins, which provide tea with a mellow taste. Compared with the other treatments, SW3 resulted in the lowest catechin content and the highest theaflavin content, suggesting that a moderate shading rate is ideal for decreasing bitterness and astringency and enhancing the mellow taste of tea infusions. Hence, RNA demethylation indirectly affects the accumulation of flavonoids, catechins, and theaflavins by triggering the AS-mediated regulatory mechanism, thereby improving the palatability of oolong tea.

**Figure 7:**
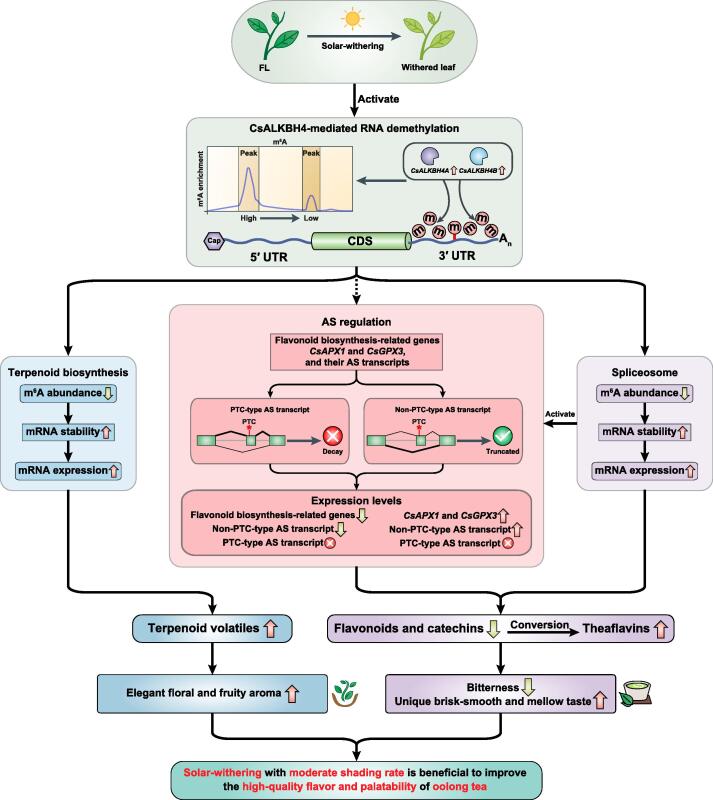
**Schematic model for the effects of m^6^A-mediated regulatory mechanism on the accumulation of flavor metabolites in tea (*Camellia sinensis*) leaves under solar-withering** CsALKBH4-driven RNA demethylation can not only directly affect the accumulation of volatile terpenoids and the tea aroma formation by mediating the stability and abundance of terpenoid biosynthesis-related genes, but also indirectly regulate the contents of flavonoids, catechins, and theaflavins, as well as the tea taste formation via triggering the AS-mediated regulatory mechanism. These findings uncover a novel layer of epitranscriptomic gene regulation in tea flavor-related metabolic pathways and establish a strong link between m^6^A-mediated regulatory mechanism and the improvement of high-quality flavor and palatability in oolong tea. Solid arrows indicate direct regulation, and dashed arrows indicate indirect regulation. PTC, premature stop codon.

In conclusion, our integrated RNA methylome and transcriptome analysis reveal that the m^6^A-mediated regulatory mechanism coordinates the accumulation of specialized metabolites in tea leaves during the solar-withering stage. Moreover, the dynamic changes in global m^6^A level that occur in tea leaves in response to different shading rates during the solar-withering step are mainly controlled by m^6^A erasers. Furthermore, CsALKBH4-driven RNA demethylation directly affects the accumulation of volatile terpenoids and tea aroma formation by mediating the stability and abundance of terpenoid biosynthesis-related transcripts, while also indirectly regulating the contents of flavonoid, catechin, and theaflavin as well as the formation of tea taste-related substances by activating the AS-mediated regulatory mechanism. These findings have elucidated the effects of epigenetic modifications on the transcription of genes in the tea flavor-related metabolic pathways. They also indicate that the m^6^A-mediated regulatory mechanism may be targeted to enhance the high-quality flavor and palatability of oolong tea (Figure 7). Our work provides a solid foundation for future attempts at deciphering the functional effects of m^6^A modifications in tea plants, and it has also broadened our understanding of the regulatory mechanisms underlying m^6^A-mediated flavor formation during the solar-withering stage of the tea manufacturing process.

## Materials and methods

### Plant materials and solar-withering treatments

Fresh tea buds with the first three leaves were collected from *C. sinensis* cv. Tieguanyin plants cultivated in a tea plantation at Fujian Agriculture and Forestry University, Fuzhou, China (E 119°14′, N 26°05′). The harvested tea leaves were evenly divided into five groups. The first group (*i.e.*, FL) was untreated and analyzed immediately. The other four groups were laid out on bamboo sieves for the solar-withering treatments with different shading rates. Briefly, black nylon sunshade nets were positioned 0.2 m above the bamboo sieves. The solar-withering treatments were as follows: SW1 (high shading rate provided by a sunshade net with 1500 meshes; 10,000 ± 400 lx); SW2 (moderate shading rate provided by a sunshade net with 1000 meshes; 20,000 ± 800 lx); SW3 (low shading rate provided by a sunshade net with 500 meshes; 40,000 ± 1200 lx); and SW4 (no sunshade net; 80,000 ± 2000 lx). The duration of the solar-withering treatment was 45 min. The other environmental parameters were consistent with those used in our previous study [24]. Three independent biological replicates were included in each solar-withering treatment. The light intensity and spectrum were measured using a spectral irradiance colorimeter (Catalog No. SPIC-300, Everfine Corporation, Hangzhou, China), whereas the UV intensity was recorded using a UV radiometer (Catalog No. UV340B, Sanpometer Corporation, Shenzhen, China). After the solar-withering treatments, the samples were collected, frozen immediately in liquid nitrogen, and stored at −80 °C.

### Quantitative analysis of the m^6^A/A ratio

The global m^6^A/A ratio for the tea leaves was determined as previously described [59]. Briefly, RNA was extracted from each sample using the TransZol UP reagent (Catalog No. ET111, TransGen, Beijing, China). Then, the quantitative analysis of the global m^6^A/A ratio was performed using the EpiQuik m^6^A RNA methylation quantification kit (Catalog No. P-9005, Epigentek, Farmingdale, NY).

### MeRIP-seq and data analysis

**Supplementary Figure S14:**
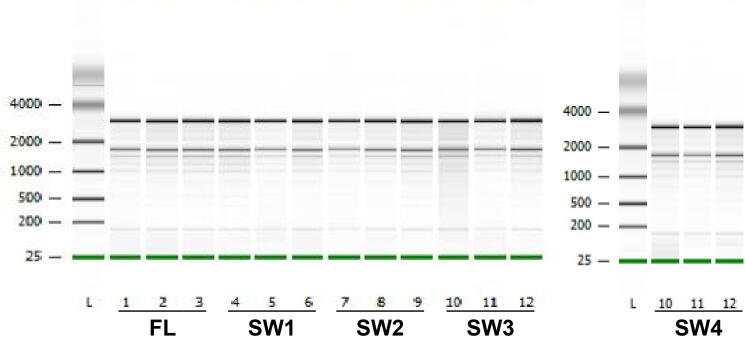
**RNA quality of fresh leaves and four solar-withered leaves with different shading rates** Three independent biological replicates were performed for each sample. FL, fresh leaves; SW1, solar-withering with a high shading rate; SW2, solar-withering with a middle shading rate; SW3, solar-withering with a low shading rate; SW4, solar-withering with natural sunlight.

For MeRIP-seq, total RNA was extracted from each sample using the TransZol UP reagent (Catalog No. ET111, TransGen). The integrity and quantity of the obtained RNA were assessed by RNase-free agarose gel electrophoresis and the Agilent 2100 bioanalyzer (Agilent Technologies, Palo Alto, CA). All RNA samples passed the quality checks, and there were no signs of RNA degradation (Figure S14). Next, poly(A) mRNA was isolated from the total RNA samples using the Dynabeads mRNA purification kit (Catalog No. 61,006, ThermoFisher Scientific, Waltham, CA). The enrichment of m^6^A was examined using the Magna MeRIP m^6^A kit (Catalog No. 17-10,499, Millipore, Billerica, MA). Briefly, the mRNA was fragmented into approximately 100-nt fragments using the fragmentation buffer. The fragmented RNA was divided into two groups, one of which was mixed with m^6^A-specific antibodies in the IP buffer. The other group of RNA was used as the input control. The m^6^A-containing fragments and non-IP fragments were used for constructing strand-specific libraries using the NEBNext Ultra II RNA library prep kit (Catalog No. E7770, New England Biolabs, Ipswich, MA). The average fragment size in each paired-end library was 100 ± 50 bp. Finally, m^6^A MeRIP-seq was performed using the Illumina NovaSeq 6000 platform (Illumina, San Diego, CA). Three independent biological replicates were sequenced for each sample.

The raw reads containing adapter sequences and undetermined bases were removed using the Trimmomatic tool [60], after which the high-quality reads were mapped to the chromosome-level tea genome using the HISAT software [61]. The R-package exomePeak [62] was used to identify the m^6^A peaks in each m^6^A-IP sample, with the corresponding non-IP sample serving as the background. In all three independent biological replicates, peaks with an overlap of at least 50% of their length and *P* < 0.05 were designated as high-confidence m^6^A peaks. The m^6^A peaks were visualized using the integrative genomics viewer software [63]. According to the genomic location information, the distribution of peaks in the non-overlapping mRNA regions, including the 5′ UTR, CDS, and 3′ UTR, was determined using BEDTools [64]. The MEME suite [32] and HOMER tool [33] were used to search for motifs within the m^6^A peaks. The DMPs between groups were identified using the DiffBind software [65], with |FC| ≥ 2 and *P* < 0.05 set as the thresholds. The FPKM values were calculated to represent the mRNA expression levels in the input libraries using the StringTie software [66]. Differentially expressed genes were identified (|FC| ≥ 2 and *P* < 0.05) using the R package DESeq2 [67]. The expression profiles of candidate genes (standardized FPKM values) were visualized using the TBtools software [68]. The rMATS program [69] was used to detect AS events, which were considered significant if the false discovery rate was less than 0.05. The AS transcripts were validated by reverse transcription-polymerase chain reaction (RT-PCR) assays involving specific primers (Table S12) as previously described [70]. The PCR products were monitored by agarose gel electrophoresis. Differentially expressed genes associated with AS events were defined as DAGs according to previously described criteria [58]. The KEGG enrichment analysis of DMP-associated genes and DAGs was performed using a published method [25].

### m^6^A-IP-qPCR and qRT-PCR analyses

The m^6^A-IP-qPCR analysis was performed according to an established procedure [11], with minor modifications. Briefly, the RNA samples used for the m^6^A MeRIP-seq analysis were fragmented into approximately 300-nt segments using the aforementioned fragmentation buffer. Some of the fragmented RNA samples were used for m^6^A-IP, which was completed using m^6^A-specific antibodies. The non-IP RNA was used as the input control. The m^6^A-containing RNA and non-IP RNA samples were reverse transcribed into cDNA using the TransScript first-strand cDNA synthesis SuperMix kit (Catalog No. AT301, TransGen). The m^6^A enrichment of specific mRNA regions was detected using the LightCycler 480 platform (Roche, Basel, Switzerland). The m^6^A abundance was quantified according to the 2^−ΔΔC_T_^ method [71]. The relative abundance of specific mRNA regions in the m^6^A-IP sample was first normalized against that of *CsActin* (GenBank: HQ420251), which has no obvious m^6^A-modified peak and served as an internal control, and then normalized against that for the non-IP sample.

The qRT-PCR analysis was conducted using the LightCycler 480 platform as previously described [24]. The non-fragmented RNA samples were reverse transcribed into cDNA as described above. *CsActin* was used to normalize the mRNA expression levels. Relative mRNA levels were calculated using the 2^−ΔΔC_T_^ method. The analysis was completed using three independent biological replicates. Details regarding the primers used for the m^6^A-IP-qPCR and qRT-PCR assays are provided in Table S12.

### Analysis of volatiles by GC**–**MS

The volatile compounds in tea samples were extracted and analyzed as previously described [72]. The Agilent model 7890B gas chromatograph and the 7000D mass spectrometer (Agilent Technologies) were used to detect the volatile compounds. Each assay was conducted using three replicates. The detected volatiles were identified according to the retention time and mass spectra data in the National Institute of Standards and Technology Mass Spectral Library (https://www.nist.gov/srd/nist-standard-reference-database-1a/). The relative abundance of individual volatile compounds was determined on the basis of the chromatogram peak area.

### Analysis of the metabolome by LC–MS

Metabolites were extracted and analyzed essentially as previously described [73]. Briefly, freeze-dried tea samples were ground into a powder using a tissue grinder (Catalog No. JXFSTPRP-24, Jingxin Company, Shanghai, China), after which 800 μl 70% methanol and 250 µl 2′,7′-dichlorofluorescein were added to the powdered material. After centrifuging the extracts at 14,000 *g* for 15 min, the supernatants were passed through a 0.22-µm polyvinylidene fluoride filter and analyzed using the ACQUITY two-dimensional ultra-performance liquid chromatography platform (Waters, Milford, MA) connected to a Q-Exactive quadrupole-orbitrap mass spectrometer (ThermoFisher Scientific). The compounds were separated in the Hypersil GOLD aQ column (100 mm × 2.1 mm, 1.9 μm, ThermoFisher Scientific), with 0.1% formic acid in pure water (v/v; solvent A) and 0.1% formic acid in acetonitrile (v/v; solvent B) used at a flow rate of 0.3 ml/min. The gradient elution was completed as follows: 5% solvent B for 2 min, linear increase to 95% solvent B over 22 min, 95% solvent B for 5 min, and then return to the initial condition (5% solvent B) within 3 min. The column temperature was set at 40 °C. Peaks were detected and the retention time was corrected using the compound discoverer software (version 3.1; ThermoFisher Scientific). The detected metabolites were identified by comparing their molecular mass, retention time, and mass spectrometry fragmentation patterns with those of the authentic standards, mzCloud (https://www.mzcloud.org/), and mzVault (https://mytracefinder.com/tag/mzvault/) databases. The relative abundance of each metabolite was calculated using the metaX tool [74]. The total flavonoid content of tea leaves was measured according to the aluminum chloride colorimetric method [24]. Three independent biological replicates were included in each experiment.

### Gene suppression and mRNA stability assays

The gene suppression assay was performed as previously described [14]. The freshly detached tea bud and the first leaf from tea plants grown under natural conditions were added to 1.5-ml microcentrifuge tubes containing 1 ml 20 μM siRNA solution or negative control siRNA (siRNA-NC) solution. After incubating for 12 h and 24 h, the tea bud and first leaf were collected for a qRT-PCR analysis. Specific siRNAs were obtained from GenePharma (Shanghai, China). Details regarding the siRNA and siRNA-NC solutions are listed in Table S12. The overall m^6^A abundance and metabolite content in the gene-silenced leaves and the NC-leaves were examined as described above.

To assess mRNA stability, leaf discs were obtained from the gene-silenced tea leaves and then immersed in sterile water that was supplemented with 10 μg/ml actinomycin D (Catalog No. A1410, Sigma, St Louis, MO). Tea leaves immersed in sterile water were used as the controls. Total RNA was isolated from the leaves sampled at 6 h and 12 h, respectively. The mRNA stability was determined using a previously described qRT-PCR method [59] and specific primers (Table S12).

### Statistical analysis

The correlations among the full-length transcripts, AS transcripts, and flavor metabolites were determined using the Pearson’s correlation coefficient. The differences among various groups were assessed by conducting a one-way analysis of variance (ANOVA) and Tukey’s *post hoc* test, with *P* < 0.05 set as the threshold for significance. Data are herein presented as the mean ± standard deviation (SD). The raw data for the qRT-PCR analysis are provided in Table S11.

## Data availability

The raw sequencing data have been deposited in the Genome Sequence Archive [75] at the National Genomics Data Center, Beijing Institute of Genomics, Chinese Academy of Sciences / China National Center for Bioinformation (GSA: CRA006400), and are publicly accessible at https://ngdc.cncb.ac.cn/.

## Conmpeting interests

The authors have declared no competing interests.

## CRediT authorship contribution statement

**Chen Zhu:** Methodology, Validation, Formal analysis, Investigation, Writing – original draft, Writing – review & editing. **Shuting Zhang:** Software, Formal analysis, Data curation, Writing – original draft. **Chengzhe Zhou:** Validation, Investigation. **Caiyun Tian:** Validation, Investigation. **Biying Shi:** Software, Data curation. **Kai Xu:** Validation, Investigation. **Linjie Huang:** Validation, Investigation. **Yun Sun:** Software, Data curation. **Yuling Lin:** Conceptualization, Formal analysis. **Zhongxiong Lai:** Conceptualization, Formal analysis, Writing – review & editing, Funding acquisition, Project administration. **Yuqiong Guo:** Conceptualization, Methodology, Formal analysis, Investigation, Writing – review & editing, Project administration. All authors have read and approved the final manuscript.

## Supplementary material

Supplementary Table S1 **A summary of m^6^A-seq reads in tea leaves under solar-withering with different shading rates**

Supplementary Table S2 **The number of m^6^A peaks detected in m6A-marked genes**

Supplementary Table S3 **Statistics of DMPs under solar-withering with different shading rates**

Supplementary Table S4 **DMP-associated genes involved in ribosome pathway**

Supplementary Table S5 **DMP-associated genes involved in RNA transport pathway**

Supplementary Table S6 **DMP-associated genes involved in spliceosome pathway**

Supplementary Table S7 **DMP-associated genes involved in the terpenoid biosynthesis pathway**

Supplementary Table S8 **Statistics of AS events under solar-withering with different shading rates**

Supplementary Table S9 **Statistics of DAGs under solar-withering with different shading rates**

Supplementary Table S10 **DAGs involved in the flavonoid biosynthesis pathway**

Supplementary Table S11 **Raw data for qRT-PCR**

Supplementary Table S12 **Primers used in this study**

## References

[1] Scarrow M., Chen N., Sun G.L. (2020). Insights into the *N*^6^-methyladenosine mechanism and its functionality: progress and questions. Crit Rev Biotechnol.

[2] Meyer K.D., Jaffrey S.R. (2014). The dynamic epitranscriptome: *N*^6^-methyladenosine and gene expression control. Nat Rev Mol Cell Biol.

[3] Fu Y., Dominissini D., Rechavi G., He C. (2014). Gene expression regulation mediated through reversible m^6^A RNA methylation. Nat Rev Genet.

[4] Wang X., Lu Z.K., Gomez A., Hon G.C., Yue Y.N., Han D.L. (2014). *N*^6^-methyladenosine-dependent regulation of messenger RNA stability. Nature.

[5] Pendleton K.E., Chen B.B., Liu K.Q., Hunter O.V., Xie Y., Tu B.P. (2017). The U6 snRNA m^6^A methyltransferase METTL16 regulates SAM synthetase intron retention. Cell.

[6] Shen L.S., Liang Z., Gu X.F., Chen Y., Teo Z.W., Hou X.L. (2016). *N*^6^-methyladenosine RNA modification regulates shoot stem cell fate in *Arabidopsis*. Dev Cell.

[7] Hu J.Z., Manduzio S., Kang H.S. (2019). Epitranscriptomic RNA methylation in plant development and abiotic stress responses. Front Plant Sci.

[8] Scutenaire J., Deragon J.M., Jean V., Benhamed M., Raynaud C., Favory J.J. (2018). The YTH domain protein ECT2 is an m^6^A reader required for normal trichome branching in *Arabidopsis*. Plant Cell.

[9] Yang D.D., Xu H.C., Liu Y., Li M.Z., Ali M., Xu X.Y. (2021). RNA *N*^6^-methyladenosine responds to low-temperature stress in tomato anthers. Front Plant Sci.

[10] Dominissini D., Moshitch-Moshkovitz S., Schwartz S., Salmon-Divon M., Ungar L., Osenberg S. (2012). Topology of the human and mouse m^6^A RNA methylomes revealed by m^6^A-seq. Nature.

[11] Zhou L.L., Tian S.P., Qin G.Z. (2019). RNA methylomes reveal the m^6^A-mediated regulation of DNA demethylase gene *SIDML2* in tomato fruit ripening. Genome Biol.

[12] Liu G.F., Wang J., Hou X.L. (2020). Transcriptome-wide *N*^6^-methyladenosine (m^6^A) methylome profiling of heat stress in pak-choi (*Brassica rapa* ssp. *Chinensis*). Plants.

[13] Zhou L.E., Tang R.K., Li X.J., Tian S.P., Li B.B., Qin G.Z. (2021). *N*^6^-methyladenosine RNA modification regulates strawberry fruit ripening in an ABA-dependent manner. Genome Biol.

[14] Zhu C., Zhang S.T., Zhou C.Z., Xie S.Y., Chen G.W., Tian C.Y. (2021). Genome-wide investigation of *N*^6^-methyladenosine regulatory genes and their roles in tea (*Camellia sinensis*) leaves during withering process. Front Plant Sci.

[15] Zeng L.T., Zhou X.C., Su X.G., Yang Z.Y. (2020). Chinese oolong tea: an aromatic beverage produced under multiple stresses. Trends Food Sci Tech.

[16] Hu C.J., Li D., Ma Y.X., Zhang W., Lin C., Zheng X.Q. (2018). Formation mechanism of the oolong tea characteristic aroma during bruising and withering treatment. Food Chem.

[17] Zeng L.T., Watanabe N., Yang Z.Y. (2019). Understanding the biosyntheses and stress response mechanisms of aroma compounds in tea (*Camellia sinensis*) to safely and effectively improve tea aroma. Crit Rev Food Sci Nutr.

[18] Zhou Y., Zeng L.T., Liu X.Y., Gui J.D., Mei X., Fu X.M. (2017). Formation of (E)-nerolidol in tea (*Camellia sinensis*) leaves exposed to multiple stresses during tea manufacturing. Food Chem.

[19] Lin N., Liu X.Y., Zhu W.F., Cheng X., Wang X.H., Wan X.C. (2021). Ambient ultraviolet B signal modulates tea flavor characteristics via shifting a metabolic flux in flavonoid biosynthesis. J Agric Food Chem.

[20] Zhang L., Cao Q.Q., Granato D., Xu Y.Q., Ho C.T. (2020). Association between chemistry and taste of tea: a review. Trends Food Sci Tech.

[21] Li Y., Shibahara A., Matsuo Y., Tanaka T., Kouno I. (2010). Reaction of the black tea pigment theaflavin during enzymatic oxidation of tea catechins. J Nat Prod.

[22] Wang Y., Zheng P.C., Liu P.P., Song X.W., Guo F., Li Y.Y. (2019). Novel insight into the role of withering process in characteristic flavor formation of teas using transcriptome analysis and metabolite profiling. Food Chem.

[23] Ni T.C., Xu S.S., Wei Y.M., Li T.H., Jin G., Deng W.W. (2021). Understanding the promotion of withering treatment on quality of postharvest tea leaves using UHPLC-orbitrap-MS metabolomics integrated with TMT-based proteomics. LWT.

[24] Zhu C., Zhang S.T., Fu H.F., Zhou C.Z., Chen L., Li X.Z. (2019). Transcriptome and phytochemical analyses provide new insights into long non-coding RNAs modulating characteristic secondary metabolites of oolong tea (*Camellia sinensis*) in solar-withering. Front Plant Sci.

[25] Zhu C., Zhang S.T., Zhou C.Z., Chen L., Zaripov T., Zhan D.M. (2020). Integrated transcriptome, microRNA, and phytochemical analyses reveal roles of phytohormone signal transduction and ABC transporters in flavor formation of oolong tea (*Camellia sinensis*) during solar withering. J Agric Food Chem.

[26] Ai Z.Y., Zhang B.B., Chen Y.Q., Yu Z., Chen H.C., Ni D.J. (2017). Impact of light irradiation on black tea quality during withering. J Food Sci Technol.

[27] Li Y.C., He C., Yu X.L., Zhou J.T., Ran W., Chen Y.Q. (2021). Effects of red-light withering on the taste of black tea as revealed by non-targeted metabolomics and transcriptomics analysis. LWT.

[28] Yang J., Zhou X.C., Wu S.H., Gu D.C., Zeng L.T., Yang Z.Y. (2021). Involvement of DNA methylation in regulating the accumulation of the aroma compound indole in tea (*Camellia sinensis*) leaves during postharvest processing. Food Res Int.

[29] Gu D.C., Yang J., Wu S.H., Liao Y.Y., Zeng L.T., Yang Z.Y. (2021). Epigenetic regulation of the phytohormone abscisic acid accumulation under dehydration stress during postharvest processing of tea (*Camellia sinensis*). J Agric Food Chem.

[30] Xia E.H., Tong W., Hou Y., An Y.L., Chen L.B., Wu Q. (2020). The reference genome of tea plant and resequencing of 81 diverse accessions provide insights into its genome evolution and adaptation. Mol Plant.

[31] Luo G.Z., Macqueen A., Zheng G.Q., Duan H.C., Dore L.C., Lu Z.K. (2014). Unique features of the m^6^A methylome in *Arabidopsis thaliana*. Nat Commun.

[32] Bailey T.L., Boden M., Buske F.A., Frith M., Grant C.E., Clementi L. (2009). MEME suite: tools for motif discovery and searching. Nucleic Acids Res.

[33] Heinz S., Benner C., Spann N., Bertolino E., Lin Y.C., Laslo P. (2010). Simple combinations of lineage-determining transcription factors prime *cis*-regulatory elements required for macrophage and B cell identities. Mol Cell.

[34] Wei L.H., Song P.Z., Wang Y., Lu Z.K., Tang Q., Yu Q. (2018). The m^6^A reader ECT2 controls trichome morphology by affecting mRNA stability in arabidopsis. Plant Cell.

[35] Zhang K., Zhuang X.J., Dong Z.Z., Xu K., Chen X.J., Liu F. (2021). The dynamics of *N*^6^-methyladenine RNA modification in interactions between rice and plant viruses. Genome Biol.

[36] Guo T.L., Liu C.H., Meng F.X., Hu L., Fu X.M., Yang Z.H. (2022). The m^6^A reader MhYTP2 regulates *MdMLO19* mRNA stability and antioxidant genes translation efficiency conferring powdery mildew resistance in apple. Plant Biotechnol J.

[37] Ehrnsberger H.F., Grasser M., Grasser K.D. (2019). Nucleocytosolic mRNA transport in plants: export factors and their influence on growth and development. J Exp Bot.

[38] Zheng H.X., Li S.M., Zhang X.S., Sui N. (2020). Functional implications of active *N*^6^-methyladenosine in plants. Front Cell Dev Biol.

[39] Wang B., Tseng E., Regulski M., Clark T.A., Hon T., Jiao Y.P. (2016). Unveiling the complexity of the maize transcriptome by single-molecule long-read sequencing. Nat Commun.

[40] Wang M.J., Wang P.C., Liang F., Ye Z.X., Li J.Y., Shen C. (2018). A global survey of alternative splicing in allopolyploid cotton: landscape, complexity and regulation. New Phytol.

[41] Mi X.Z., Yue Y., Tang M.S., An Y.L., Xie H., Qiao D.H. (2021). TeaAS: a comprehensive database for alternative splicing in tea plants (*Camellia sinensis*). BMC Plant Biol.

[42] Zhang G.Y., Yang J.H., Cui D.D., Zhao D.D., Benedito V.A., Zhao J. (2020). Genome-wide analysis and metabolic profiling unveil the role of peroxidase CsGPX3 in theaflavin production in black tea processing. Food Res Int.

[43] Zhang G.Y., Yang J.H., Cui D.D., Zhao D.D., Li Y.Y., Wan X.C. (2020). Transcriptome and metabolic profiling unveiled roles of peroxidases in theaflavin production in black tea processing and determination of tea processing suitability. J Agric Food Chem.

[44] Tong W., Li R.P., Huang J., Zhao H.J., Ge R.H., Wu Q. (2021). Divergent DNA methylation contributes to duplicated gene evolution and chilling response in tea plants. Plant J.

[45] Wang L., Shi Y., Chang X.J., Jing S.L., Zhang Q.J., You C.J. (2018). DNA methylome analysis provides evidence that the expansion of the tea genome is linked to TE bursts. Plant Biotechnol J.

[46] Nelson D.C., Flematti G.R., Riseborough J.A., Ghisalberti E.L., Dixon K.W., Smith S.M. (2010). Karrikins enhance light responses during germination and seedling development in *Arabidopsis thaliana*. Proc Natl Acad Sci U S A.

[47] Collings E.R., Alamar M.C., Márquez M.B., Kourmpetli S., Kevei Z., Thompson A.J. (2021). Improving the tea withering process using ethylene or UV-C. J Agric Food Chem.

[48] Reape T.J., Molony E.M., Mccabe P.F. (2008). Programmed cell death in plants: distinguishing between different modes. J Exp Bot.

[49] Shamala L.F., Zhou H.C., Han Z.X., Wei S. (2020). UV-B induces distinct transcriptional re-programing in UVR8-signal transduction, flavonoid, and terpenoids pathways in *Camellia sinensis*. Front Plant Sci.

[50] Shao C.Y., Zhang C.Y., Lv Z.D., Shen C.W. (2021). Pre- and post-harvest exposure to stress influence quality-related metabolites in fresh tea leaves (*Camellia sinensis*). Sci Hortic.

[51] Huong T.T., Ngoc L.N., Kang H.S. (2020). Functional characterization of a putative RNA demethylase ALKBH6 in *Arabidopsis* growth and abiotic stress responses. Int J Mol Sci.

[52] Xu Z.H., Shi X.B., Bao M.M., Song X.Q., Zhang Y.X., Wang H.Y. (2021). Transcriptome-wide analysis of RNA m^6^A methylation and gene expression changes among two *Arabidopsis* ecotypes and their reciprocal hybrids. Front Plant Sci.

[53] Zhang F., Zhang Y.C., Liao J.Y., Yu Y., Zhou Y.F., Feng Y.Z. (2019). The subunit of RNA *N*^6^-methyladenosine methyltransferase OsFIP regulates early degeneration of microspores in rice. PLoS Genet.

[54] Duan H.C., Wei L.H., Zhang C., Wang Y., Chen L., Lu Z. (2017). ALKBH10B is an RNA *N*^6^-methyladenosine demethylase affecting *Arabidopsis* floral transition. Plant Cell.

[55] Zheng H.X., Sun X., Li J.L., Song Y.S., Song J., Wang F. (2021). Analysis of *N*^6^-methyladenosine reveals a new important mechanism regulating the salt tolerance of sweet sorghum. Plant Sci.

[56] Chen M.X., Zhang K.L., Zhang M., Das D., Fang Y.M., Dai L. (2020). Alternative splicing and its regulatory role in woody plants. Tree Physiol.

[57] Dvinge H. (2018). Regulation of alternative mRNA splicing: old players and new perspectives. FEBS Lett.

[58] Li Y.Y., Mi X.Z., Zhao S.Q., Zhu J.Y., Guo R., Xia X.B. (2020). Comprehensive profiling of alternative splicing landscape during cold acclimation in tea plant. BMC Genomics.

[59] Hu J.Z., Cai J., Park S.J., Lee K., Li Y.X., Chen Y. (2021). *N*^6^-methyladenosine mRNA methylation is important for salt stress tolerance in *Arabidopsis*. Plant J.

[60] Bolger A.M., Lohse M., Usadel B. (2014). Trimmomatic: a flexible trimmer for Illumina sequence data. Bioinformatics.

[61] Kim D., Langmead B., Salzberg S.L. (2015). HISAT: a fast spliced aligner with low memory requirements. Nat Methods.

[62] Meng J., Lu Z.L., Liu H., Zhang L., Zhang S.W., Chen Y.D. (2014). A protocol for RNA methylation differential analysis with MeRIP-seq data and exomePeak R/Bioconductor package. Methods.

[63] Thorvaldsdóttir H., Robinson J.T., Mesirov J.P. (2013). Integrative genomics viewer (IGV): high-performance genomics data visualization and exploration. Brief Bioinform.

[64] Quinlan A.R., Hall I.M. (2010). BEDTools: a flexible suite of utilities for comparing genomic features. Bioinformatics.

[65] Stark R., Brown G. (2011). DiffBind: differential binding analysis of ChIP-seq peak data. Bioconductor.

[66] Pertea M., Pertea G.M., Antonescu C.M., Chang T.C., Mendell J.T., Salzberg S.L. (2015). StringTie enables improved reconstruction of a transcriptome from RNA-seq reads. Nat Biotechnol.

[67] Love M.I., Huber W., Anders S. (2014). Moderated estimation of fold change and dispersion for RNA-seq data with DESeq2. Genome Biol.

[68] Chen C.J., Chen H., Zhang Y., Thomas H.R., Frank M.H., He Y.H. (2020). TBtools: an integrative toolkit developed for interactive analyses of big biological data. Mol Plant.

[69] Shen S.H., Park J.W., Lu Z.X., Lin L., Henry M.D., Wu Y.N. (2014). rMATS: robust and flexible detection of differential alternative splicing from replicate RNA-Seq data. Proc Natl Acad Sci U S A.

[70] Xu Q.S., Zhu J.Y., Zhao S.Q., Hou Y., Li F.D., Tai Y.L. (2017). Transcriptome profiling using single-molecule direct RNA sequencing approach for in-depth understanding of genes in secondary metabolism pathways of *Camellia sinensis*. Front Plant Sci.

[71] Livak K.J., Schmittgen T.D. (2001). Analysis of relative gene expression data using real-time quantitative PCR and the 2^−ΔΔC_T_^ method. Methods.

[72] Zhang W.J., Cao J.X., Li Z.G., Li Q.H., Lai X.F., Sun L.L. (2021). HS-SPME and GC/MS volatile component analysis of Yinghong No. 9 dark tea during the pile fermentation process. Food Chem.

[73] Yu X.M., Xiao J.J., Chen S., Yu Y., Ma J.Q., Lin Y.Z. (2020). Metabolite signatures of diverse *Camellia sinensis* tea populations. Nat Commun.

[74] Wen B., Mei Z.L., Zeng C.W., Liu S.Q. (2017). MetaX: a flexible and comprehensive software for processing metabolomics data. BMC Bioinformatics.

[75] Chen T.T., Chen X., Zhang S.S., Zhu J.W., Tang B.X., Wang A.K. (2021). The Genome Sequence Archive Family: toward explosive data growth and diverse data types. Genomics Proteomics Bioinformatics.

